# *Oviductus Ranae* alleviates D-galactose-induced ovarian aging by inhibiting ferroptosis and regulating the GPX4/ACSL4 pathway

**DOI:** 10.1186/s13048-025-01857-2

**Published:** 2025-11-28

**Authors:** Xiaomei Ling, Cong Xie, Ming Li, Jie Li, Shun Liu, Liyue Sun, Xinxin Zeng, Zhen Zhang, Xuhui Zhang, Lei Liang

**Affiliations:** 1 Medical Research Center, The Affiliated Guangdong Second Provincial General Hospital of Jinan University, Guangzhou, 510317 PR China; 2 Department of Pediatrics, The Second Affiliated Hospital of Guangzhou Medical University, Guangzhou, 510260 PR China; 3 Department of Health Management Centre, Zhongshan Hospital, Fudan University, Shanghai, 200032 PR China; 4 Department of Oncology, The Affiliated Guangdong Second Provincial General Hospital of Jinan University, Guangzhou, 510317 PR China; 5 The Second School of Clinical Medicine, Southern Medical University, Guangzhou, 510515 PR China; 6 Guangdong Engineering Research Center of Chinese Medicine & Disease Susceptibility/ School of Traditional Chinese Medicine, Jinan University, Guangzhou, 510632 PR China

**Keywords:** *Oviductus Ranae*, Ovarian aging, Ovarian granulosa cell, Ferroptosis, Lipid peroxidation

## Abstract

**Background:**

Ovarian dysfunction caused by aging restricts female reproductive capacity and causes age-related health problems. Ferroptosis is an important mode of cell death during accelerated aging. *Oviductus Ranae* (OR), a traditional Chinese medicine, has been used to treat ovarian age-related diseases in women. However, the mechanisms through which OR mitigates ovarian aging, especially ferroptosis regulation, remain unclear. This study investigated the pharmacological effects and mechanisms of OR for ovarian aging in rats.

**Methods:**

Sprague–Dawley female rats were divided into six groups: aging group induced with D-galactose; experiment groups treated with OR at low, middle, and high concentrations; positive control treated with estradiol valerate; and control group with no treatment. After 42 d, ovarian tissue and serum were collected for biochemical determination, hematoxylin and eosin staining, immunohistochemistry, enzyme-linked immunosorbent assay, reverse transcription-quantitative polymerase chain reaction, western blotting, and RNA sequencing analysis. To elucidate the mechanism underlying the effects of OR on ferroptosis in ovarian granulosa cells (OGCs), in vitro experiments were conducted.

**Results:**

Treatment with OR improved ovarian follicular development and serum hormone levels, reduced iron deposition, enhanced cell proliferation, and inhibited apoptosis in D-galactose-induced ovarian aging rats. Ferroptosis was alleviated in the ovaries and serum by elevated glutathione and decreased lipid peroxidation production. RNA-seq showed that OR induced changes in 2,768 genes that were involved in ovarian steroidogenesis, glutathione metabolism, lipid metabolism, and ferroptosis pathways. Notably, OR downregulated ACSL4, LPCAT3, and ALOX12 mRNA and protein expressions, while upregulating FTH1, FTL, SLC7A11, GSS, and GPX4, which serve as critical regulators in ferroptosis. OGCs pretreated with OR-containing serum before erastin exposure exhibited enhanced viability, reduced ferrous iron, total iron, and lipid peroxidation levels, improved antioxidant activity, and stabilized mitochondrial function, indicating effective ferroptosis inhibition. Furthermore, OR-containing serum pretreatment inhibited ferroptosis in OGCs by regulating the GPX4/ACSL4 axis.

**Conclusions:**

OR resists ovarian damage and prevents aging through antiferroptosis mechanisms by regulating the GPX4/ACSL4 axis in OGCs and aging rats. These results highlight the potential of OR as a therapeutic agent for the prevention and treatment of ovarian aging.

**Supplementary Information:**

The online version contains supplementary material available at 10.1186/s13048-025-01857-2.

## Introduction 

Ovarian aging begins early in life and progresses rapidly, with ovarian functions generally declining sharply at the age of 35 years. Normal ovarian aging includes follicle and oocyte degeneration, causing estrogen insufficiency and the onset of menopause [1]. Ovarian aging impacts menstrual cycles, fertility, and mental disorders; increases the risk of obstetric and gynecological complications, osteoporosis, and cardiovascular diseases; and contributes to the aging process of other organs, severely impacting female physical and mental health. Most symptoms of ovarian aging are closely associated with hormone deficiency. Therefore, hormone replacement therapy (HRT) is typically used to manage symptoms of ovarian aging and prevent related diseases. However, this treatment modality may increase the risk of cancer, stroke, and cardiovascular diseases [2]. Although the mechanisms underlying age-related ovarian dysfunction remain unclear, they primarily involve the antioxidant system and free radicals, mitochondrial dysfunction, altered telomeres and telomerase activity, and cell apoptosis [3]. Moreover, although several studies have reported on menopause, menopausal syndrome, and premature ovarian failure [4–6], they have not drawn direct parallels with ovarian aging. Hence, elucidating the mechanisms underlying ovarian aging is essential for identifying safer alternative treatments.

Ovarian aging is a central factor in female aging, and ferroptosis has been identified as an important mode of cell death during accelerated aging [7]. As a unique type of cell death, ferroptosis is regulated by iron-induced oxidative damage [8]. This complex process involves iron-driven phospholipid oxidation and is influenced by diverse cellular metabolic pathways, such as those involving iron regulation, redox balance, and mitochondrial function; and lipid, amino acid, and glycan metabolism [9]. Hence, considering that ferroptosis is closely related to the damage and degeneration of various organs [10], its regulation holds considerable promise for treating ischemic organ injuries, drug-resistant cancers, and other degenerative diseases related to extensive lipid peroxidation [11]. Mechanistically, ferroptosis participates in the mediation of several vital genes that influence cellular pathophysiological processes. For instance, glutathione peroxidase 4 (GPX4) is a selenoenzyme that plays a central role in impeding ferroptosis by using glutathione (GSH) as a cofactor to convert lipid hydroperoxides into non-toxic lipid alcohols [12]. In addition, the cystine-glutamate antiporter system, comprising SLC7A11 and SLC3A2, is a key regulatory factor in iron-induced cell death that can be suppressed by erastin. Inhibiting this system reduces cystine uptake, which can trigger ferroptosis [12]. In addition, ferritin serves as a major intracellular iron storage protein and an essential element of the cellular antioxidant defense system [13]. Although progress has been made in elucidating the impact of ferroptosis on female reproduction, particularly in ovarian cancer [14], endometriosis [15], and PCOS [13], the impact of ferroptosis on ovarian aging remains unclear. Therefore, understanding the regulatory mechanisms underlying iron-induced cell death may prove critical for developing new therapeutic methods for ovarian aging.

Traditional Chinese medicine (TCM) is commonly used for complementary or alternative therapies in menstrual disorders, perimenopausal symptoms, and infertility, with positive outcomes in the treatment of ovarian aging [5, 16–18]. In TCM theory, the kidney is considered the foundation of reproductive health, and kidney deficiency is regarded as the root cause of ovarian aging. The TCM *Oviductus Ranae* (OR) is derived from the dried oviduct of female frogs (*Rana temporaria chensinensis David*) with the ability to tonify the kidney and essence, reconcile yin, and moisten the lungs [19]. Accordingly, OR is commonly administered to address a wide range of conditions related to kidney deficiency, including perimenopausal syndrome, menopause, estrogen deficiency-induced osteoporosis, and reproductive aging [20]. OR can delay reproductive aging in female mice by increasing estrogen levels in elderly mice, prolonging the estrous cycle, reducing malondialdehyde (MDA) levels, and promoting superoxide dismutase (SOD) activity, thus enhancing the antioxidant effects in the ovaries and uterus. Ultimately, the ovarian and uterine indices and pathological changes are improved with OR [21]. In addition, OR exerts a protective effect on ovarian granulosa cells (OGCs) by reducing reactive oxygen species (ROS) production, inhibiting mitochondrial membrane depolarization, downregulating pro-apoptotic markers (Bax, caspase-3, caspase-9, and p53), and upregulating the anti-apoptotic protein Bcl-2. OR also activates the MAPK signaling pathway, contributing to resistance against lipid peroxidation and cell apoptosis [22]. However, the role of OR in regulating ferroptosis during ovarian aging has not yet been explored.

D-galactose is a well-known reducing sugar that has often been used in aging models. D-galactose may induce an excessive accumulation of advanced glycation products in oxidative metabolism, leading to cell and tissue senescence [23]. The D-galactose-induced ovarian aging model closely resembles the natural aging process in animals, making it useful in constructing aging ovarian models [24]. 

Therefore, in this study, D-galactose was used to establish the rat model of ovarian aging and investigate the protective effects of OR on the ovaries, and erastin-stimulated OGCs were used to elucidate the underlying mechanisms of action.

## Materials and methods

### Reagents

OR was obtained from the Guangzhou Miaoyingtang Medicinal Materials Store (Guangzhou, China). Primers were synthesized by Guangzhou IGE Biotechnology, Ltd. (Guangzhou, China).

The following reagents and compounds were used throughout the study: estradiol valerate (EV; lot no. 629A; Guandong Branch of Bayer Healthcare Co., Ltd., Guangzhou, China), D-galactose (Cat. no. G100369; Shanghai Aladdin Biochemical Co., Ltd., Shanghai, China), erastin and ferrostatin-1 (Fer-1; Selleck Chemicals, Shanghai, China), pregnant mare serum gonadotropin (Hangzhou Animal Medicine Factory, Hangzhou, China), tetramethylrhodamine ethyl ester perchlorate (TMRE; MedChem Express, Monmouth Junction, NJ, USA), and rhodamine 123 (Beyotime Biotechnology, Shanghai, China).

The following kits were used: cell counting kit-8 (CCK-8) and annexin V/propidium iodide (Yeasen Biotechnology Co., Ltd., Shanghai, China); FerroOrange (Dojindo Molecular Technologies Inc., Shanghai, China); C11 BODIPY 581/591 (GLPBIO, Shanghai, China); total iron (Fe), ROS, MDA, and GSH assay kits; DAB (SA-HRP) transferase dUTP nick end labeling (TUNEL) cell apoptosis detection kit; Prussian blue staining kit; and hematoxylin and eosin (H&E) kit (Servicebio, Wuhan, China). Enzyme-linked immunosorbent assay (ELISA) kits for anti-Müllerian hormone (AMH; LV20844), estradiol (E_2_; LV20525), progesterone (P; LV20529), follicle-stimulating hormone (FSH; LV20526), and luteinizing hormone (LH; LV20527) were obtained from Animal Union Biotechnology Co., Ltd. (Shanghai, China). Additional reagents included the IntraSure kit (BD Biosciences, NJ, USA), MolPure cell/tissue total RNA kit (Yeasen Biotechnology Co., Ltd., Shanghai, China), PrimeScript™ RT reagent kit with gDNA eraser (Cat. no. RR047A; Takara Bio, Inc., Shanghai, China), SYBR Green Taq qPCR master mix (Cat. no. A301-10), BCA protein assay kit (GenStar, Shanghai, China), and enhanced chemiluminescence assay kit (Millipore, Billerica, MA, USA).

The following antibodies were used: 4-hydroxynonenal (4-HNE; MAB3249-SP; R&D Systems, Minneapolis, MN, USA), GPX4 (NBP3-07344), xCT (NB300-318SS), and LPCAT3 (NBP3-04752; Novus Biologicals, Centennial, CO, USA), GPX4 (ab125066), FTH1 (ab183781), and ACSL4 (ab155282; Abcam, Cambridge, UK), GSS (67598), FTL (10727), and GAPDH (60004; Proteintech Group, Inc., Wuhan, China), ALOX12 (bs-3874R; Bioss, Beijing, China), proliferating cell nuclear antigen (PCNA; GB11010) and rabbit anti-goat IgG (GB23204; Servicebio, Wuhan, China), goat anti-rabbit IgG (7074S) and anti-mouse IgG (7076S; Cell Signaling Technology Inc., Danvers, MA, USA).

### Animals

Sprague–Dawley (SD) female rats weighing 200–220 g and suckling rats weighing 40–50 g, aged 23–27 d, were obtained from the Guangdong Medical Laboratory Animal Center (License Number: SCXK [Yue]: 2023-0002). Rats were maintained under controlled conditions with a 12 h dark/light cycle, a temperature range of 24–26 °C, and *ad libitum* access to food and water. All experimental operations complied with the Regulations on the Management of Laboratory Animals issued by the Guangdong Provincial Government.

### Animal model of D-galactose-induced ovarian aging

SD female rats were randomly divided into six groups: control; model; low- (L), medium- (M), and high- (H) -dose OR; and positive control with EV (*n* = 8 rats/group). To determine the apt dosage of OR and EV for rats, proportionately relative to human doses, the body surface area normalization method was used [25]. The following equation was used to determine the equivalent daily dosage for rats, designated as D2: D2 = D1 × 5.36 g/kg. In this equation, D1 signifies the dosage meant for human consumption. This foundational medium dosage constituted the standard for further dosing considerations. The low and high dosages were derived as 0.5- and 2-fold of the amount of the initial medium dose in that order. In practice, the conventional dosage for adult humans (10 g/60 kg) was translated to derive the rat medium dosage. The conversion formula used was the following: D2 = (10 g ÷ 60 kg) × 5.36, resulting in a 0.9 g/kg equivalent for rats. Thus, the OR-L, -M, and -H groups were respectively administered 0.45, 0.9, and 1.8 g/kg OR suspension through gavage, equivalent to 0.5-, 1-, and 2-fold the human dose. The EV group received 0.18 mg/kg EV suspension. Excluding the control group, rats in the model, OR, and EV groups were subcutaneously injected with D-galactose (350 mg/kg) on the back of the neck once daily for 6 weeks, consecutively. The control group received subcutaneous injections of physiological saline. Starting from the third week, OR and EV were prepared in vegetable oil and administered through gavage for 4 weeks, whereas the model and control groups received equivalent doses of vegetable oil, also through gavage.

Rats were anesthetized and euthanized; their ovaries and blood were collected for subsequent analysis, and the body weight of the rats as well as the wet weight of the ovaries were measured (ovarian index = ovarian weight/body weight, mg/g).

### H&E staining

Ovarian tissues were fixed in 4% paraformaldehyde (PFA) for 48 h. The ovarian tissue wax block was obtained through gradient ethanol dehydration, transparency, wax immersion, and embedding. After the ovarian wax block was sliced into 4-µm-thick slices, the ovary sections were stained using H&E and observed using a light microscope (AXIOSCOPE.A1, Zeiss, Oberkochen, Germany).

### Serum hormone levels

ELISA kits were used to determine AMH, FSH, LH, E_2_, and P levels in rat serum samples. A microplate reader (ELx800, BioTek, USA) was used to detect the absorbance at 450 nm.

### Prussian blue staining

Paraffin-embedded ovarian sections were deparaffinized, rehydrated, and stained with Perls staining solution for 40 min. After washing with pure water for 20–30 s, the slices were lightly stained with eosin staining solution for 3 min, followed by rinsing with pure water for 20–30 s. Finally, the stained slices were dehydrated with anhydrous ethanol, made transparent with xylene, sealed with neutral glue, and observed using a light microscope.

### PCNA staining

Paraffin-embedded ovarian sections were deparaffinized and rehydrated. Subsequently, the endogenous peroxidase activity was quenched by immersing in methanol containing 0.3% hydrogen peroxide. After heating for 20 min at 100 °C in citrate buffer (pH 6.0) for antigen retrieval, the sections were blocked with goat serum for 20 min. The anti-PCNA antibody (1:1000) was added and incubated at 4 °C overnight, followed by treatment with a goat anti-mouse secondary antibody (1:200) at 25 °C in the dark for 1 h. Finally, the sections were stained with a DAB peroxidase substrate kit and hematoxylin, and observed and photographed using a light microscope.

### TUNEL staining

Paraffin-embedded ovarian sections were deparaffinized, rehydrated, and stained using the TUNEL Apoptosis Detection Kit (DAB Colorimetric Method), according to the manufacturer’s instructions. After the incubation period, images of the slides were examined using a light microscope.

### Serum biochemical indicators

Total Fe, MDA, and GSH levels in rat serum were determined following the kit instructions. After serum treatment, absorbance values at 593, 532, and 412 nm were determined using a microplate reader (BioTek, USA).

### Immunohistochemistry (IHC)

Paraffin-embedded ovarian sections were deparaffinized and rehydrated, after which the endogenous peroxidase activity was quenched by immersing in methanol containing 0.3% hydrogen peroxide. After heating for 20 min at 100 °C in citrate buffer (pH 6.0) for antigen retrieval, the sections were blocked with 5% goat serum and incubated with primary antibody against 4-HNE (1:300) and GPX4 (1:150) at 4 ℃ overnight. Subsequently, the sections were incubated with a goat anti-mouse secondary antibody (1:200) for 50 min at 25 °C in the dark, followed by staining using a DAB peroxidase substrate kit and hematoxylin, then observed and photographed using a light microscope. Image-Pro Plus 6.0 software was used for semi-quantification. Staining was quantified by measuring the immunoreactive area (IA) in µm^2^ and the integrated optical density (IOD). The staining intensity (SI) for each image was calculated as SI = IOD/IA.

### Preparation of drug-containing rat serum for in vitro experiments

The contents of OR powder and EV tablets were ground in a mortar and suspended in vegetable oil solution. OR drug-containing serum was prepared following a previously established method [22]. Briefly, the OR dose for an adult woman (10 g/60 kg) was converted to the low dose for a rat (0.9 g/kg). The medium and high dosages were calculated as 3- and 9-fold the initial low dose, respectively. Thus, the OR-L, OR-M, and OR-H groups were given 0.90, 2.70, and 8.10 g/kg OR through gavage, respectively, which is equivalent to 1-, 3-, and 9-fold the human dose, and the EV group received 0.54 mg/kg EV suspension, whereas rats of the control group were given the same volume of vegetable oil daily. Each group was administered the intervention or control over 7 consecutive days. Blood samples were collected from the abdominal aorta 1.5 h after the final gavage and then centrifuged at 3000 rpm for 15 min to separate the serum, which was subsequently sterilized through a 0.22-µm filtration membrane. Next, the serum samples were heated with water at 56 °C for 30 min and then stored at − 80 °C for further cellular experiments.

### Primary ovarian granulosa cell (OGC) collection and culture

Twenty female SD rats, aged 25 d, were intraperitoneally injected with 60 IU of pregnant mare serum gonadotropin and sacrificed through neck dissection after 48 h. The ovaries were aseptically dissociated and placed in pre-cooled sterile PBS. Subsequently, the surrounding marginal tissues were removed from the ovaries. Follicles were punctured with a 1-mL syringe needle to release the OGCs into DMEM/F12. OGCs were passed through a 40-µm sterile filter and collected through centrifuged at 400 × *g* for 5 min. A single-cell suspension was prepared by adding DMEM/F12 supplemented with 10% FBS. Trypan blue staining was performed to count the viable cells, adjust the cell suspension concentration, and seed the cells in culture flasks. The OGCs were cultured at 37 °C in an incubator with 5% CO_2_ for 72 h, and adherent OGCs were collected for subsequent experiments. The OGCs were identified using the method reported in a previous study [26]. Briefly, OGC identification was performed via immunofluorescence staining for FSH receptor expression.

### OGC models and treatment

OGCs were divided into the following groups: control, model (erastin, 20 µM), OR-L (0.90 g/kg), OR-M (2.70 g/kg), OR-H (8.10 g/kg), EV (0.54 mg/kg), and Fer-1 (10 µM). In the control, model, and Fer-1 cell groups, normal rat serum was administered, whereas the OR-L, OR-M, OR-H, and EV cell groups received a 20% volume fraction of drug-containing serum diluent before being cultured for 48 h; this was in light of a previous study showing that a 20% concentration of OR-containing serum significantly enhances OGCs proliferation [26]. Subsequently, 20 µM of erastin was administered to all groups but the control, and the incubation period was extended to 24 h.

### Detection of cell viability

The optimal concentration and time required for detecting the effects of erastin on OGCs were investigated using a CCK-8 assay. The OGCs were seeded in a 96-well plate and pre-cultured for 24 h before being intervened with a 20% volume fraction of drug-containing serum for 48 h and subsequently exposed to 20 µM erastin for 24 h. The protective effect of OR-containing serum against erastin-induced ferroptosis in OGCs was determined using a CCK-8 assay, and the absorbance values at 450 nm were detected using an automatic microplate reader.

### Detection of dead cells

Following pretreatment with drug-containing serum and erastin exposure, OGCs were collected. The OGCs were washed and stained in the dark with 0.3 µg/mL DAPI for 5 min, and the rate of dead cells was detected using a flow cytometer (FACSAria™ Ⅲ, Becton Dickinson, NJ, USA).

### Fe assay

Total Fe level was determined using an iron assay kit. The absorbance value at 450 nm was determined using an automatic microplate reader, and ferrous Fe was observed through FerroOrange staining. The OGC suspension was seeded into a confocal dish. After drug treatment, the OGCs were stained with 1 µmol/L FerroOrange working solution at 37 ℃ for 30 min. The OGCs were washed before being observed and imaged using a confocal microscope (Stellaris, Leica, Wetzlar, Germany).

### Measurement of ROS and lipid peroxidation

A ROS assay kit and C11 BODIPY 581/591 probe were used to determine ROS and lipid peroxidation levels in OGCs. Briefly, the OGCs were incubated with 5 µM DCFH-DA or 2 µM C11 BODIPY for 30 min, then washed and analyzed using a flow cytometer (FACSAria™ III, Becton Dickinson, NJ, USA). The mean fluorescence intensities of DCF and C11 BODIPY in the FITC-A channel were recorded.

### Determination of OGC antioxidant capacity

OGCs were seeded in a six-well plate. After drug intervention, the OGCs were washed and resuspended in 50 µL PBS. Subsequently, 100 µL of Reagent A was added and treated in the dark for 5 min. After washing, 50 µL of Reagent B containing antibodies (4-HNE, 1:300; GPX4, 1:200) was added and incubated in the dark for 20 min. Next, OGCs were washed and stained with 100 µL of Alexa Fluor 488 fluorescent secondary antibody (1:400) for 40 min at 25 °C. Subsequently, the OGCs were washed, collected, and resuspended in PBS, and the average fluorescence intensities of 4-HNE and GPX4 were detected through flow cytometry.

### Mitochondrial membrane potential (MMP) detection

Rhodamine 123 staining was used to detect MMP levels in OGCs. Initially, OGCs were seeded in a six-well plate. After drug treatment, OGCs were collected, washed, and treated with 10 mmol/L rhodamine 123 solution at 37 °C for 30 min. Finally, the average fluorescence intensity was determined using a flow cytometer.

Observation and analysis of MMP changes in OGCs were performed using TRME staining. OGCs were seeded in confocal dishes and pre-cultured for 24 h before treatment with drug-containing serum and erastin. After drug intervention, OGCs were washed and stained with 500 nM TRME solution at 37 °C for 30 min. Finally, the OGCs were observed and imaged using a confocal microscope.

### Immunofluorescence (IF) assay

The OGC suspension was seeded in a confocal dish. Following drug treatment and erastin exposure, the OGCs were washed and fixed in a solution of 4% PFA for 30 min, followed by permeabilization at 4 °C for 12 min. Next, the OGCs were blocked with an immunostaining blocking solution for 15 min before incubation with the anti-GPX4 antibody overnight at 4 °C. The following day, the Alexa Fluor 488 fluorescent secondary antibody (1:400) was added, incubated at 25 °C for 50 min in the dark, washed thrice with PBS, and treated with DAPI-containing anti-fluorescence quenching and sealing solution. Finally, the stained OGCs were observed using a confocal microscope.

### RNA sequencing (RNA-Seq) analysis

Total RNA was extracted from the ovaries with TRIzol for transcriptomic analysis. The library construction, high-throughput sequencing, and data analysis were carried out by Guangzhou IGE Biotechnology, Ltd. (Guangzhou, China). Additionally, Gene Ontology (GO) functional enrichment and Kyoto Encyclopedia of Genes and Genomes (KEGG) pathway enrichment analyses were conducted using the clusterProfiler package in R.

### Reverse transcription-quantitative polymerase chain reaction (RT-qPCR)

Total RNA was extracted from the ovaries and OGCs using a cell/tissue total RNA kit. Total RNA was quantified and reverse-transcribed using a PrimeScript™ RT reagent kit with a gDNA eraser. The cDNA products were subjected to analysis using RT-qPCR with SYBR Green Taq qPCR master mix. Cyclophilin A was used as an internal reference to measure the relative mRNA content. The primer sequences used in this study are listed in Table 1.

**Table 1:** Primers used in this study for RT-qPCR

Primer name	Forward sequence (5’–3’)	Reverse sequence (5’–3’)
Cyclophilin A	TCAACCCCACCGTGTTCTTC	TCCTTTCTCCCCAGTGCTCA
SLC7A11	TAACCTTTTGCAAGCTCACAGC	AGGGCAACCCCATTAGATTTGT
GSS	TCCAAAGCCCTGAAACAGATTG	GGTTGTTGGACAGGATCTTGGA
GPX4	GAATTCGCAGCCAAGGACATC	CACGTTGGTGACGATGCACAC
FTH1	AGAGGGAACATGCTGAGAAACT	ATTCACACTCTTTTCCAAGTGC
FTL1	CTCCTCAAGTTGCAGAACGAAC	CCAGGGTTTTACCCCACTCATC
ACSL4	AGGATATGATGCCCCTCTTTGT	CATGCGGACATTTCCTCCTAGA
LPCAT3	AGGACAGCTACCTCATCCATCT	GCACAACACATAGCAAGGAGTG
ALOX12	ACCTCAGACAATAGCAGCAGAC	TTCCAGCTTCTCAGGAGGGTAT

### Western blotting

Ovaries and OGCs were added to the RIPA lysis buffer, and total protein was extracted after lysis. The protein content was quantified using the BCA method. After protein electrophoresis using sodium dodecyl sulfate–polyacrylamide gel electrophoresis (SDS-PAGE), the proteins were transferred to a polyvinylidene difluoride (PVDF) membrane and subsequently blocked using a protein-free rapid blocking solution for 10 min. The PVDF membrane was washed with TBST for 10 s and incubated with primary antibodies specific for SLC7A11, GSS, GPX4, FTH1, FTL, ACSL4, LPCAT3, and ALOX12 (1:3,000 dilution for all) overnight at 4 °C, followed by treatment with appropriate secondary antibodies at 25 °C for 1 h. After another round of washing, an enhanced chemiluminescence solution was used for visualization. Protein band intensity was quantified using the ImageJ software.

### Statistical analysis

Continuous variables were presented as mean ± standard deviation (SD). Differences among multiple groups were analyzed using one-way analysis of variance (ANOVA) followed by a least significant difference (LSD) post hoc test. Statistical analyses were performed using SPSS (version 22.0). Statistical significance was set at *P* < 0.05.

## Results

### OR can partially restore ovarian function in rats with D-galactose-induced ovarian aging

**Fig. 1:**
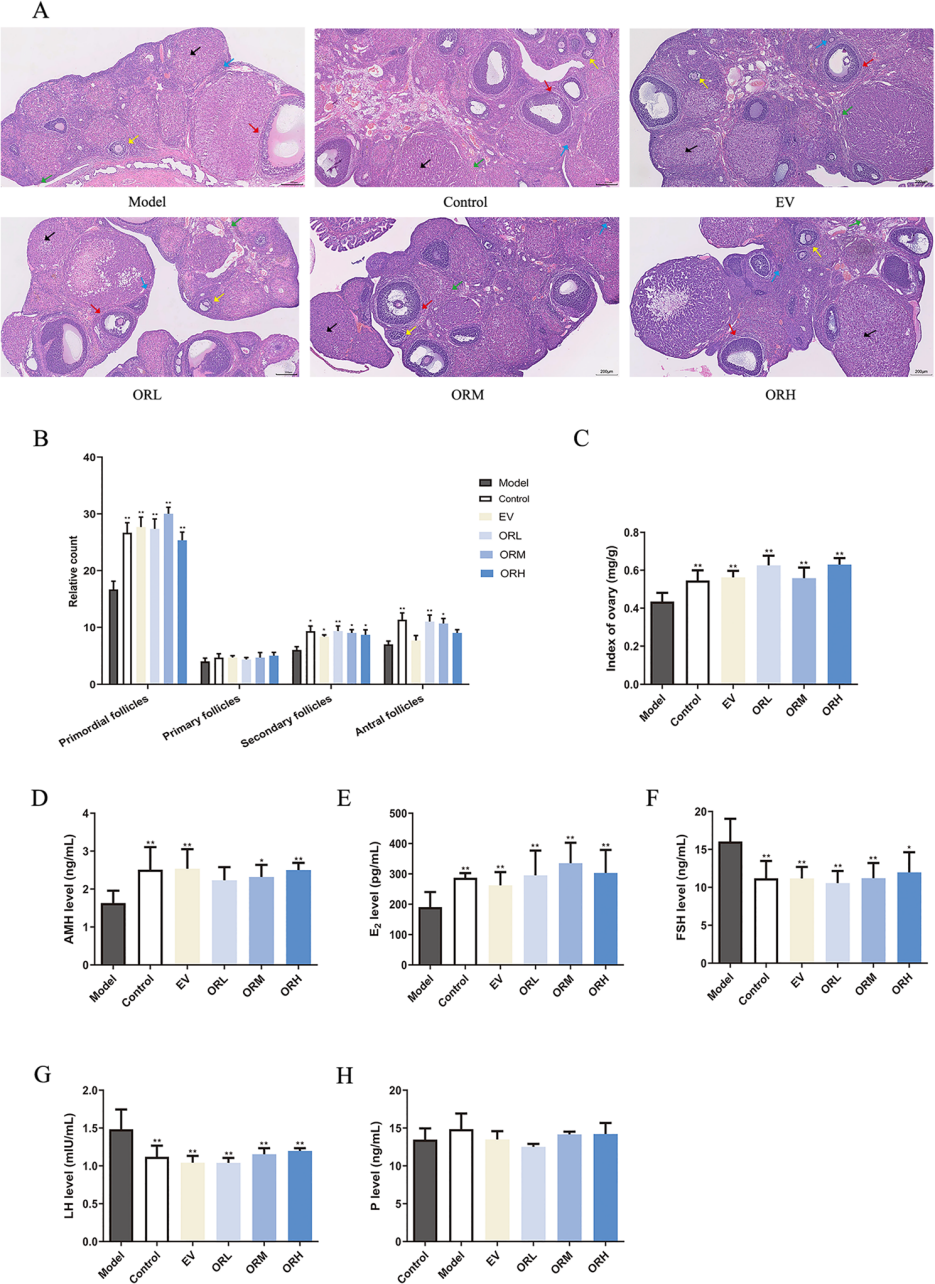
OR can partially restore ovarian function in D-galactose-induced ovarian aging rats. **(A)** H&E staining in ovarian tissues. Green, blue, yellow, and red arrows represent primordial, primary, secondary, and antral follicles, respectively. Black arrows represent the corpus luteum. Scale bar, 200 μm. **(B)** Count of primordial, primary, secondary, and antral follicles of each group. **(C)** Ovarian index (ovarian/body weight). (**D**–**H)** Serum hormone levels in rats, detected using ELISA. Results are expressed as mean ± SD for each group (*n* = 3). *P < 0.05, **P < 0.01 compared with the model group

The histological characteristics of follicles at each stage after OR treatment are shown in Fig. 1A. The ovaries in the control rats were well-developed, with follicles in different stages and detectable corpora lutea. In contrast, the D-galactose model group exhibited decreased primordial, secondary, and antral follicles in the ovaries (Fig. 1B). The numbers of primordial, secondary, and antral follicles in the OR-L and OR-M groups were higher than those in the D-galactose model group, whereas the EV group only experienced an increase in the number of primordial and secondary follicles. The number of primary follicles and corpora lutea did not differ in each group. Thus, OR may promote the development of secondary follicles to antral and mature follicles in aging rats. In addition, the ovarian index was obviously reduced in the D-galactose group and recovered significantly after supplementation with either EV or low, medium, or high OR doses (Fig. 1C).

Rat serum E_2_, FSH, LH, and P levels were measured *via* ELISA to evaluate ovarian endocrine function. In addition, serum AMH levels were used to assess ovarian reserve function. Serum E_2_ and AMH levels in the D-galactose model group were markedly lower than those in the control group; serum FSH and LH levels in the D-galactose group were significantly higher than those in the control group; and serum P levels did not differ compared to those of the control group. Notably, compared with the D-galactose model group, serum AMH and E_2_ levels in the EV and OR groups were increased (Fig. 1D and E), and serum FSH and LH levels in the EV and OR groups exhibited a decreasing trend; however, serum P level did not differ significantly (Fig. 1F–H). These results indicate that OR improved serum sex hormone and AMH levels, as well as ameliorated damage to aging ovarian tissue morphology and growing follicles in the D-galactose-induced ovarian aging model.

### OR alleviates the D-galactose-induced excessive accumulation of ovarian Fe and cell apoptosis

**Fig. 2:**
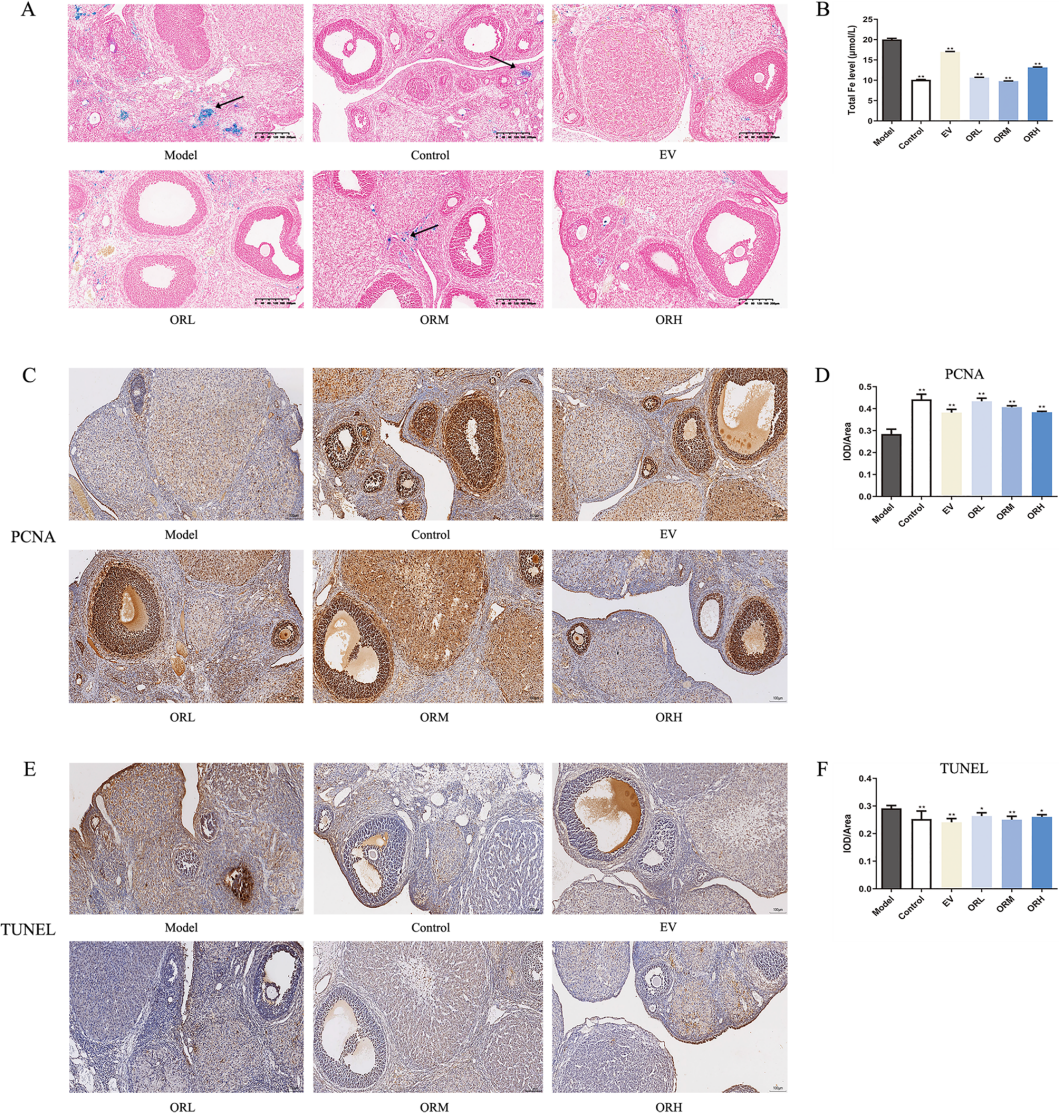
OR alleviates the D-galactose-induced excessive accumulation of ovarian Fe and cell apoptosis. **A** Prussian blue staining of ovarian tissue in each group. Arrows indicate Fe aggregation in the ovaries; Scale bar, 200 μm. **B** Total Fe levels in ovarian tissues. PCNA **(C–D)** and TUNEL **(E–F)** staining of ovarian cell proliferation and apoptosis, respectively. Scale bar, 100 μm. Results are expressed as mean ± SD for each group (*n* = 3). *P < 0.05, **P < 0.01 compared with the model group

The effect of OR on Fe accumulation in the ovarian tissue of D-galactose-induced ovarian aging rats was observed and analyzed. A notable increase in Fe deposition was detected in the D-galactose model group compared with the control group (Fig. 2A). Fe aggregation in the EV and OR groups was markedly diminished compared with the model group. A similar trend was observed for total Fe level in the ovaries (Fig. 2B).

Compared with the control group, the number of PCNA-positive cells (proliferating cells) decreased in the model group (Fig. 2C and D). Notably, the number of TUNEL-positive cells (apoptotic cells) increased (Fig. 2E and F). Furthermore, the number of proliferating cells in the EV and OR groups increased, and the number of apoptotic cells diminished compared with that of the model group. Hence, OR may reduce the excessive accumulation of Fe in the ovaries, stimulate cell proliferation, and inhibit cell apoptosis in aging rats.

### OR inhibits ferroptosis in the ovaries of rats with D-galactose-induced ovarian aging

**Fig. 3:**
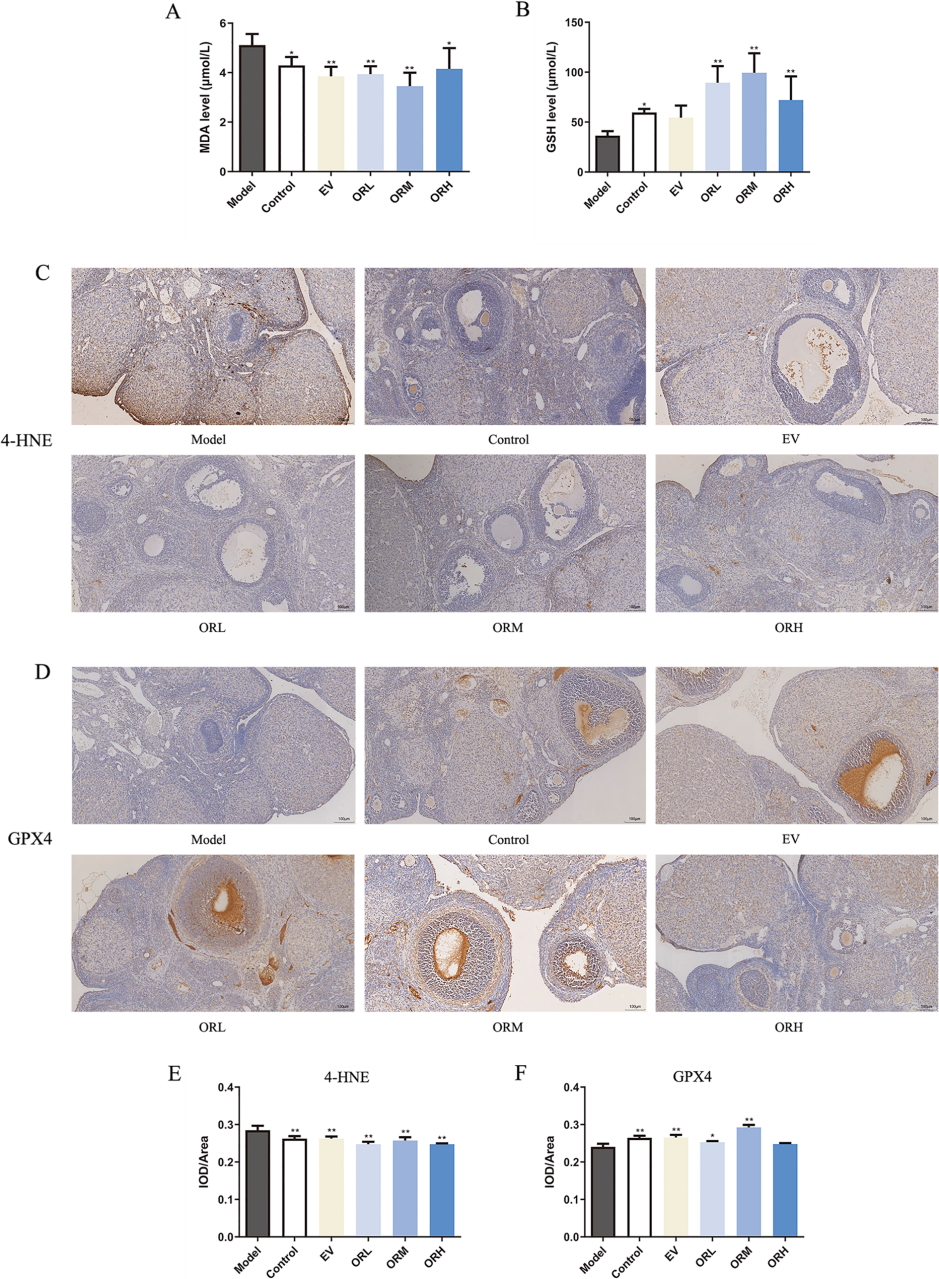
OR reduces lipid peroxidation and increases the antioxidant capacity of D-galactose-treated rat ovaries. MDA **(A)** and GSH **(B)** levels; 4-HNE **(C**, **E)** and GPX4 **(D**, **F)** IHC staining in the ovarian sections of D-galactose-treated rats. Scale bar, 100 μm. Results are expressed as mean ± SD for each group (*n* = 3). *P < 0.05, **P < 0.01 compared with the model group

Lipid peroxidation is a primary mechanism underlying ferroptosis. GPX4 and GSH homeostasis is crucial for resisting lipid peroxidation and inhibiting ferroptosis. Therefore, the effect of OR on the cellular lipid peroxidation level and antioxidant capacity of the ovary was evaluated in D-galactose-treated rats. Compared with the control, the MDA level increased, whereas the GSH level declined in the model group. The MDA levels in the EV and OR groups decreased, whereas GSH levels increased compared with the model group (Fig. 3A and B). The IHC results showed that 4-HNE expression in the ovaries of rats in the D-galactose model group was evidently higher than that in the ovaries of rats in the control group, whereas GPX4 expression was lower (Fig. 3C–F). However, after OR intervention, the expression of GPX4 in the ovaries of the OR group was evidently higher than that in the ovaries of the D-galactose model group, and the expression of 4-HNE was lower. These results indicate that OR alleviated ferroptosis in the ovaries of aging rats by reducing lipid peroxidation production and increasing the antioxidant capacity.

### RNA-Seq-based identification of differentially expressed genes (DEGs) and enrichment analysis of GO and KEGG pathways

**Fig. 4:**
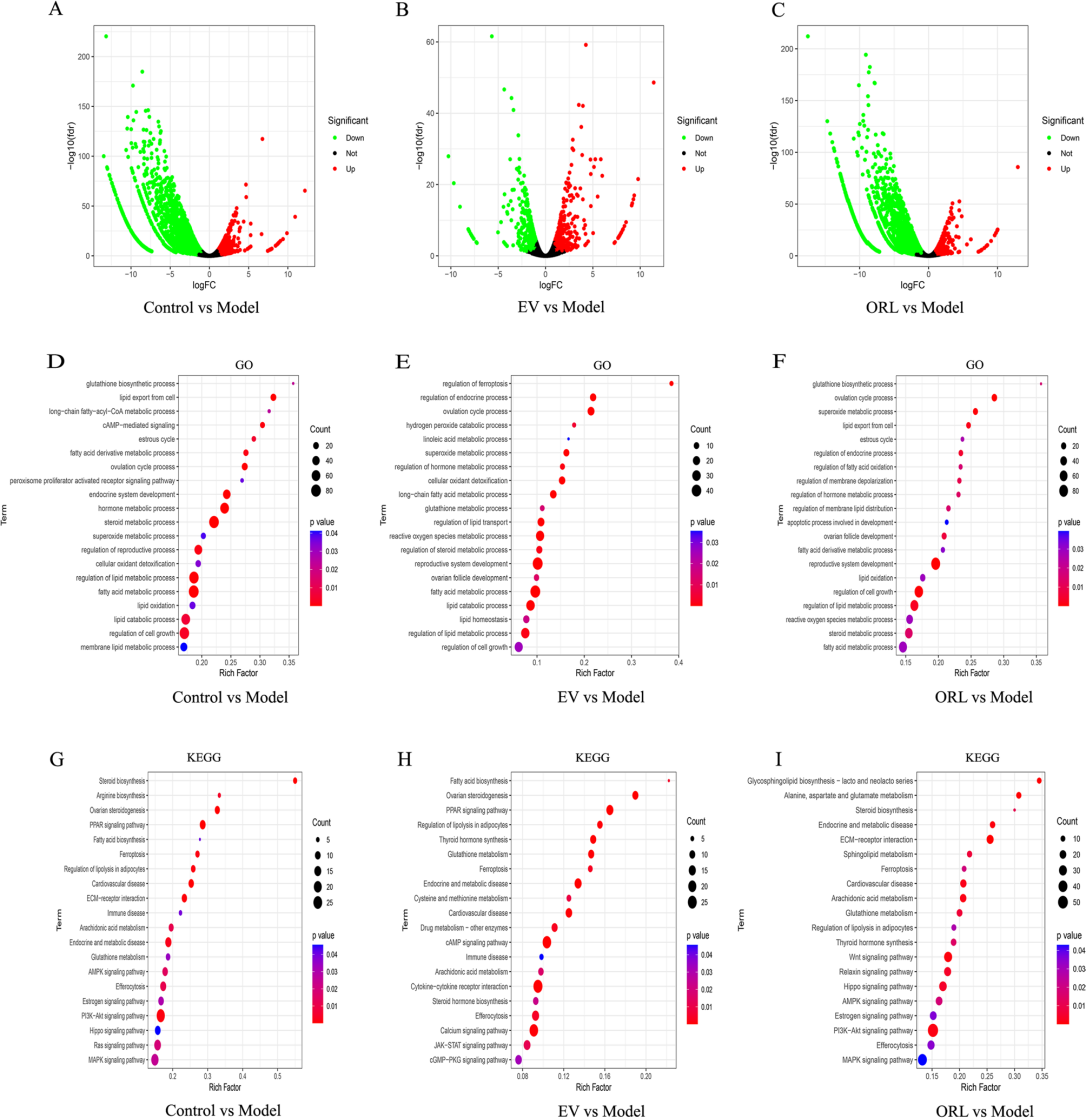
Altered gene expression and pathway enrichment analysis of aging rat ovaries following OR treatment. **A**–**C** Volcano plot of DEGs. Red and green colors represent upregulated and downregulated gene expression, respectively. **D**–**F** Biological processes enrichment. **G**–**I** KEGG pathway enrichment

To further investigate the underlying mechanisms of OR in treating ovarian aging, RNA-Seq analysis was conducted using the clusterProfiler package in R. A total of 2,975 DEGs in rat ovary tissue were identified after D-galactose stimulation, whereas 878 and 2,768 DEGs were identified in rat ovary tissue treated with EV and OR-L, respectively. Overall, 702 upregulated and 2273 downregulated DEGs were identified in the control vs. model groups, 457 upregulated and 421 downregulated DEGs were identified in the EV vs. model groups, and 479 upregulated and 2,289 downregulated DEGs were identified in the OR-L vs. model groups (Fig. 4A–C).

GO analysis showed that compared with the D-galactose model group, the DEGs in the biological process category of the control group were primarily enriched in cell growth in response to steroid, lipid, and fatty acid metabolic processes (Fig. 4D). Compared to the D-galactose model group, the DEGs in biological process of the EV and OR-L groups were correlated with regulation of reproductive system development, cell growth, and the lipid metabolic process (Fig. 4E and F).

KEGG pathway analysis showed that OR primarily affected ovarian steroidogenesis, PI3K/Akt signaling, glutathione metabolism, and ferroptosis (Fig. 4G–I). These pathways have been associated with cellular stress responses, apoptosis, proliferation, metabolism, and senescence, indicating that OR acts through these pathways to exert a therapeutic effect on ovarian aging.

### OR regulates GPX4/ACSL4 signaling to inhibit ferroptosis in ovarian aging rats

**Fig. 5:**
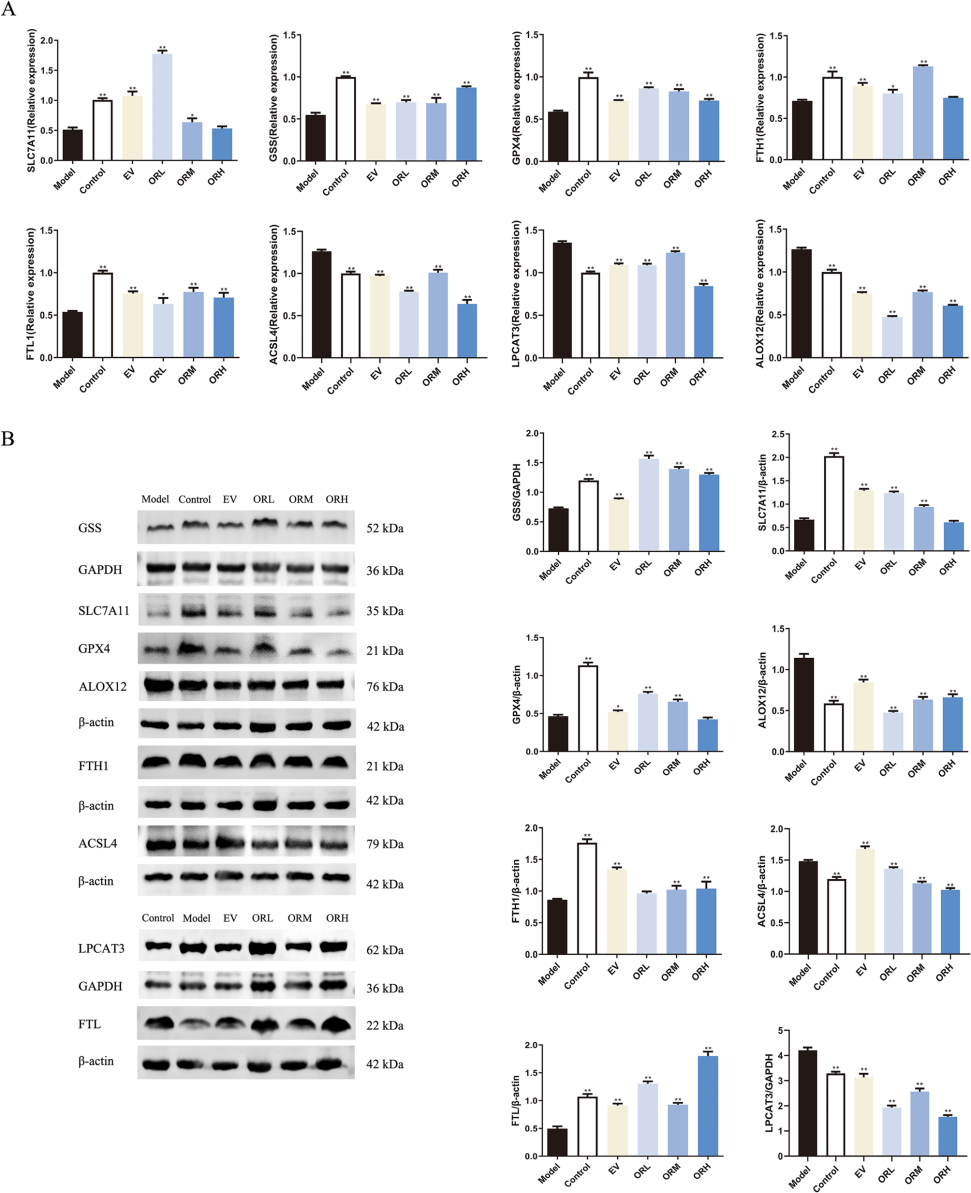
OR regulates ferroptosis-related marker expression in rats with aging ovaries. **(A)** mRNA and **(B)** protein expression of SLC7A11, GSS, GPX4, FTH1, FTL, ACSL4, LPCAT3, and ALOX12. Results are expressed as mean ± SD for each group (*n* = 3). *P < 0.05, **P < 0.01 compared with the model group

Following the enrichment analysis of the ferroptosis signaling pathway, we investigated the effect of OR on the ferroptosis pathway in ovarian aging rats. Western blotting and RT-qPCR techniques were used to evaluate SLC7A11, GSS, GPX4, ACSL4, LPCAT3, and ALOX12 mRNA and protein expression levels, following OR intervention in ovarian aging rats, which serve as critical regulators in ferroptosis. The expression of the positive regulators ACSL4, LPCAT3, and ALOX12 was upregulated in D-galactose-treated aging rats. Notably, the levels of the negative regulators SLC7A11, GSS, and GPX4 decreased. After OR intervention, the levels of ACSL4, LPCAT3, and ALOX12 decreased in the ovarian tissue, whereas SLC7A11, GSS, and GPX4 expression increased (Fig. 5A and B). Furthermore, OR upregulated FTH1 and FTL, which are involved in cytoplasmic and mitochondrial Fe storage. These results indicate that OR can partially improve ovarian function and prevent ovarian aging through anti-ferroptosis mechanisms by regulating the GPX4/ACSL4 axis in ovarian aging rats.

### OR-containing serum stimulated OGCs’ proliferation and suppressed their death

**Fig. 6:**
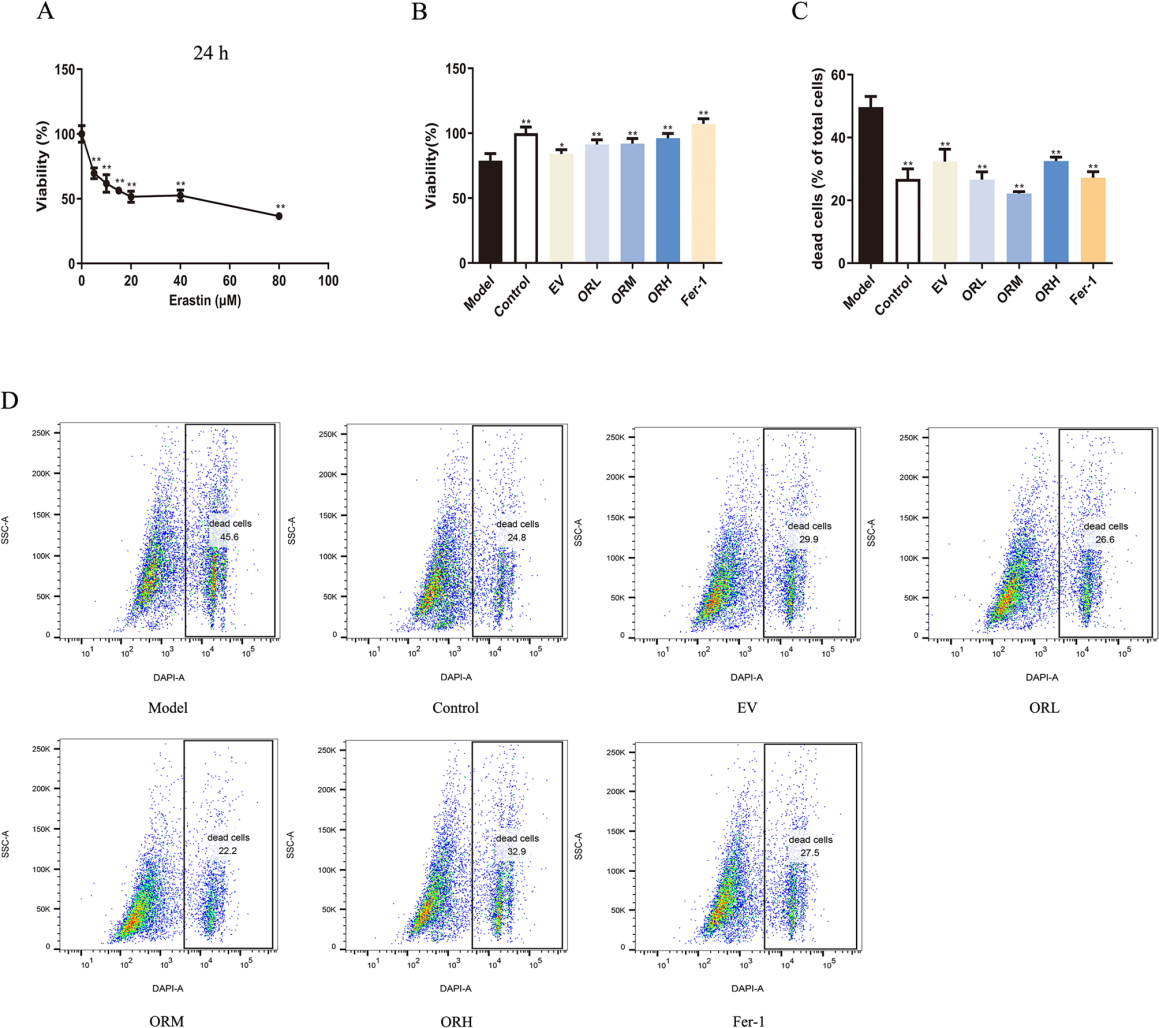
OR-containing serum stimulated proliferation and suppressed OGCs’ death. **(A)** Cell viability of OGCs treated with different concentrations of erastin for 24 h. Cell viability **(B)** and death rates **(C**–**D)** in OGCs pretreated with 20% OR-containing serum for 48 h and 20 µM erastin for 24 h. Results are expressed as mean ± SD for each group (*n* = 3). *P < 0.05, **P < 0.01 compared with the model group

To confirm whether OR-containing serum has a protective effect against ferroptosis, we investigated its effect on ferroptosis in rat OGCs stimulated with erastin in vitro. Following 20 µM erastin treatment, the viability of OGCs decreased to 51.59 ± 4.25% (Fig. 6A). Therefore, this concentration was selected to establish the OGC ferroptosis model.

Erastin induced OGC ferroptosis and reduced cell viability, whereas pretreatment with serum containing different concentrations of OR significantly improved OGC viability (Fig. 6B). DAPI staining combined with flow cytometry revealed that the death rate of the erastin model group was 49.70 ± 3.39%, whereas the death rates of the pretreatment groups with different concentrations of the OR-containing serum (L, M, H), EV-containing serum, and the ferroptosis inhibitor (Fer-1) decreased to 26.57 ± 2.50%, 22.17 ± 0.59%, 32.53 ± 1.25%, 32.33 ± 3.96%, and 27.20 ± 1.93% (Fig. 6C and D). Thus, OR-containing serum in OGCs stimulates their proliferation, inhibits their death, and exerts a protective effect against erastin-induced ferroptosis.

### OR-containing serum inhibits erastin-induced ferroptosis in OGCs

**Fig. 7:**
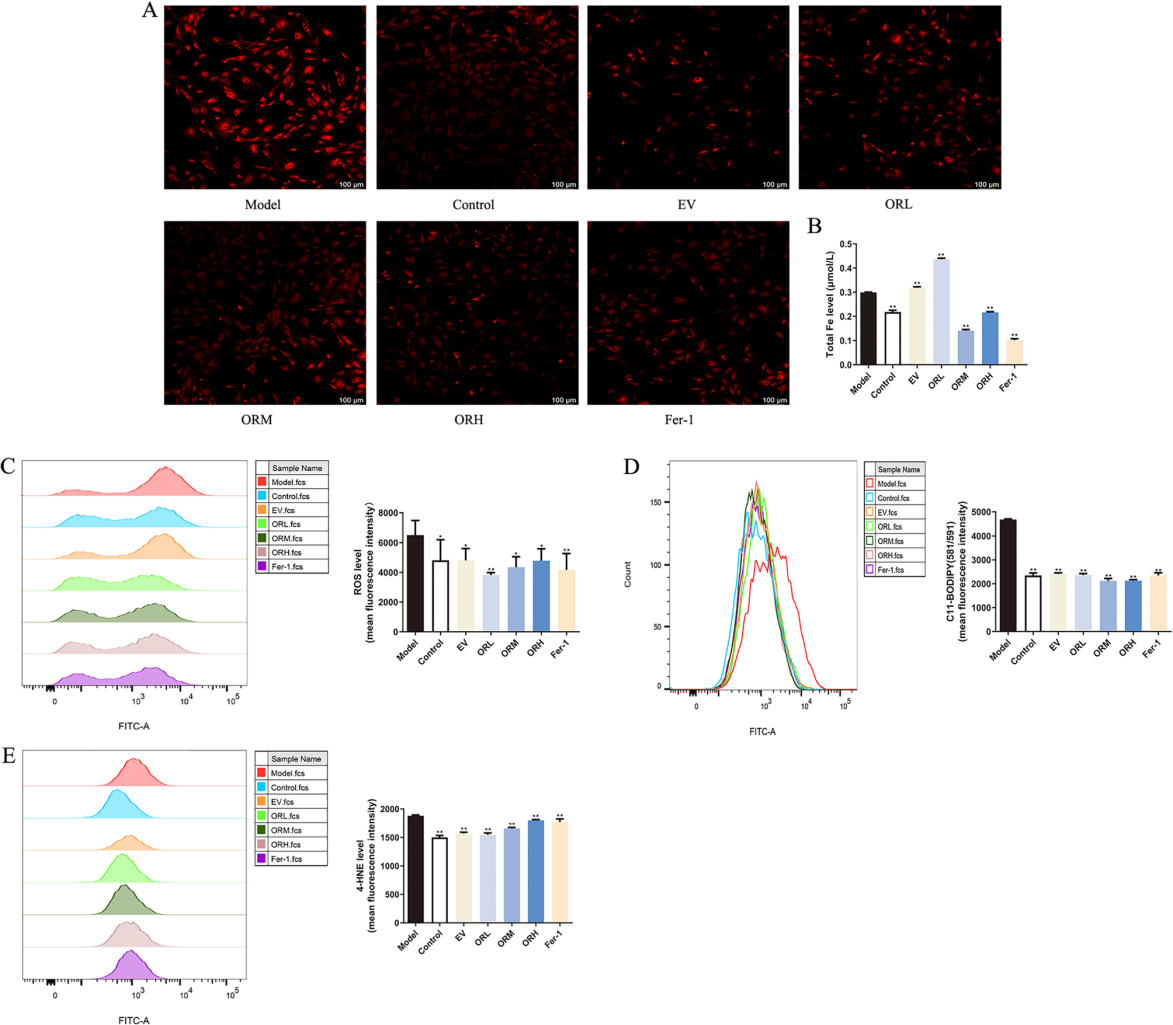
OR-containing serum inhibits erastin-induced ferroptosis in OGCs. Ferrous Fe **(A)** and total Fe **(B)** levels in OGCs. Scale bar = 100 μm. Mean fluorescence intensities of ROS **(C)** and oxidation-C11 BODIPY **(D)** in OGCs. **(E)** Levels of 4-HNE in OGCs. Results are expressed as mean ± SD for each group (*n* = 3). *P < 0.05, **P < 0.01 compared with the model group

The effect of OR-containing serum on OGC ferroptosis was evaluated. The pathophysiological mechanisms underlying ferroptosis include Fe accumulation and lipid peroxidation. Ferrous Fe (Fig. 7A) and total Fe levels (Fig. 7B) were markedly elevated in erastin-treated OGCs. After OR-containing serum intervention, ferrous Fe and total Fe levels were significantly diminished in OGCs.

The mean fluorescence intensities of ROS and oxidation-C11 BODIPY were augmented in the model groups compared with the control group and reduced following Fer-1, EV-, and OR-containing serum treatment. Furthermore, OR-containing serum and Fer-1 showed similar inhibitory effects on ROS production (Fig. 7C) and lipid peroxidation (Fig. 7D) in erastin-treated OGCs. Similarly, erastin exposure stimulated 4-HNE production in OGCs, whereas OR-containing serum and Fer-1 had the opposite effect (Fig. 7E). These results indicated that OR-containing serum treatment alleviated ferroptosis in erastin-treated OGCs by reducing the excessive accumulation of Fe and lipid peroxides.

### OR-containing serum elevates GPX4 activity and stabilizes mitochondrial function in erastin-treated OGCs

**Fig. 8:**
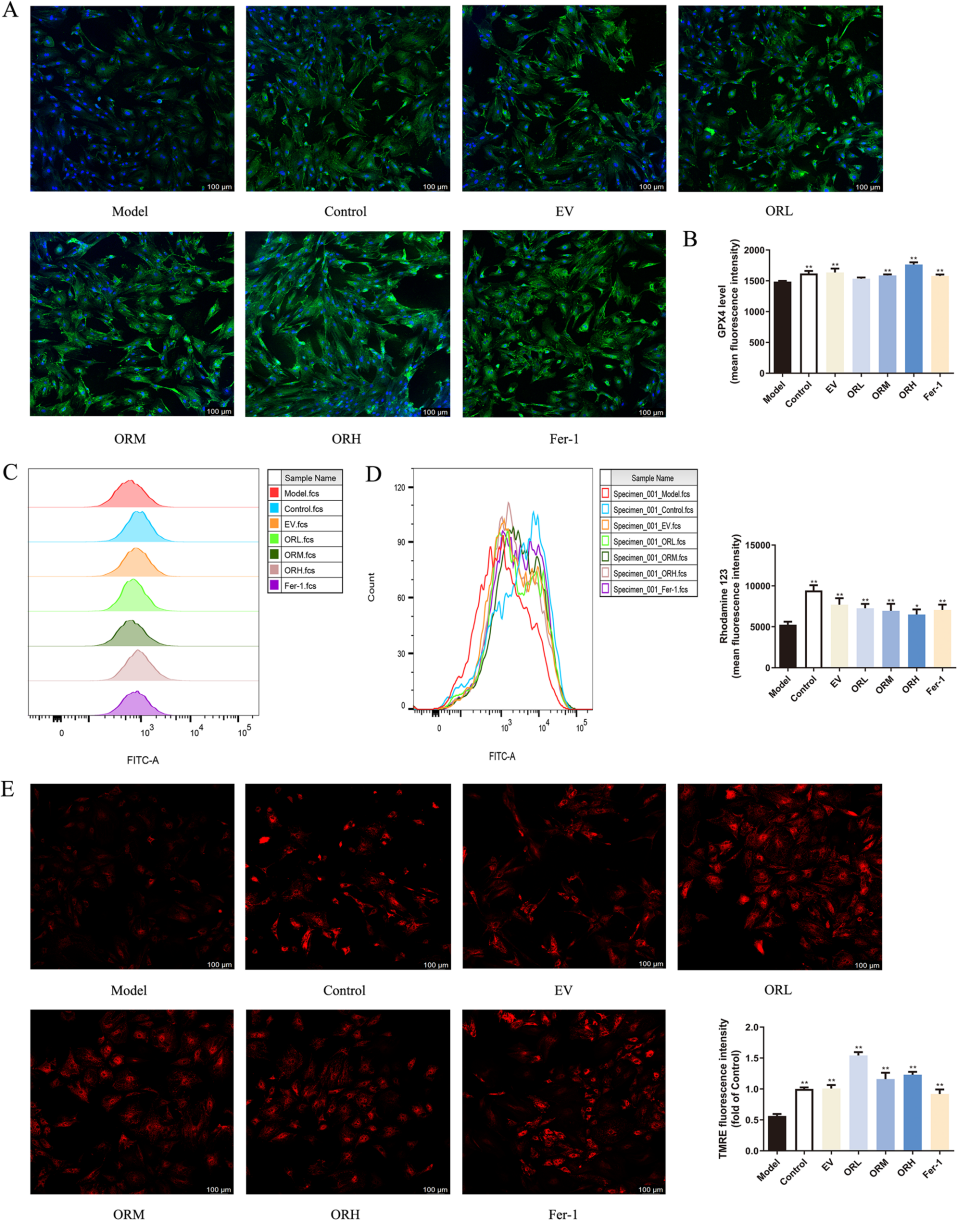
OR-containing serum elevates GPX4 activity and stabilizes mitochondrial function in erastin-treated OGCs. GPX4 expression assessed using IF **(A)** and flow cytometry **(B–C)**. Scale bar = 100 μm. Rhodamine 123 **(D)** and TMRE **(E)** staining of mitochondrial membrane potential levels; scale bar = 100 μm. Results are expressed as mean ± SD for each group (*n* = 3). *P < 0.05, **P < 0.01 compared with the model group

SLC7A11 and GPX4 downregulation revealed that erastin activated ferroptosis. GPX4 is an important regulator of ferroptosis, and SLC7A11 acts as an upstream mediator of GPX4. To further confirm whether erastin-activated ferroptosis was inhibited by OR-containing serum in OGCs, we determined the protein levels of GPX4 using IF and flow cytometry. IF (Fig. 8A) and flow cytometry (Fig. 8B and C) revealed decreased GPX4 expression in the erastin model group and significantly increased expression in the Fer-1, EV-, and OR-containing serum groups. Moreover, pretreatment with OR-containing serum ameliorated the impact of erastin-induced GPX4 decreased expression dose-dependently (Fig. 8B and C). In addition, we observed that an increase in GPX4 expression was accompanied by distinct morphological features in the OR-H group (Fig. 8A). These features may be associated with the effect of OR-containing serum in stimulating the proliferation and differentiation of OGCs. These results suggest that OR-containing serum may inhibit ferroptosis in OGCs by enhancing SLC7A11 and suppressing GSH depletion, thereby elevating GPX4 activity.

We assessed the effects of OR-containing serum on the cellular MMP of rat OGCs. Reduced MMP—an indicator of mitochondrial dysfunction—is generally related to enhanced ROS generation. Therefore, rhodamine 123 was combined with flow cytometry to analyze MMP levels (Fig. 8D), and TMRE staining was used in combination with confocal microscopy to observe changes in the MMP of OGCs (Fig. 8E). Following erastin exposure, the red fluorescence intensity, corresponding to MMP level, decreased markedly in OGCs. However, OR-containing serum inhibited the decrease in MMP, enhancing the cellular fluorescence intensity. These results indicated that OR-containing serum protected OGCs against erastin-induced mitochondrial dysfunction.

### OR-containing serum regulates the GPX4/ACSL4 axis to inhibit ferroptosis

**Fig. 9:**
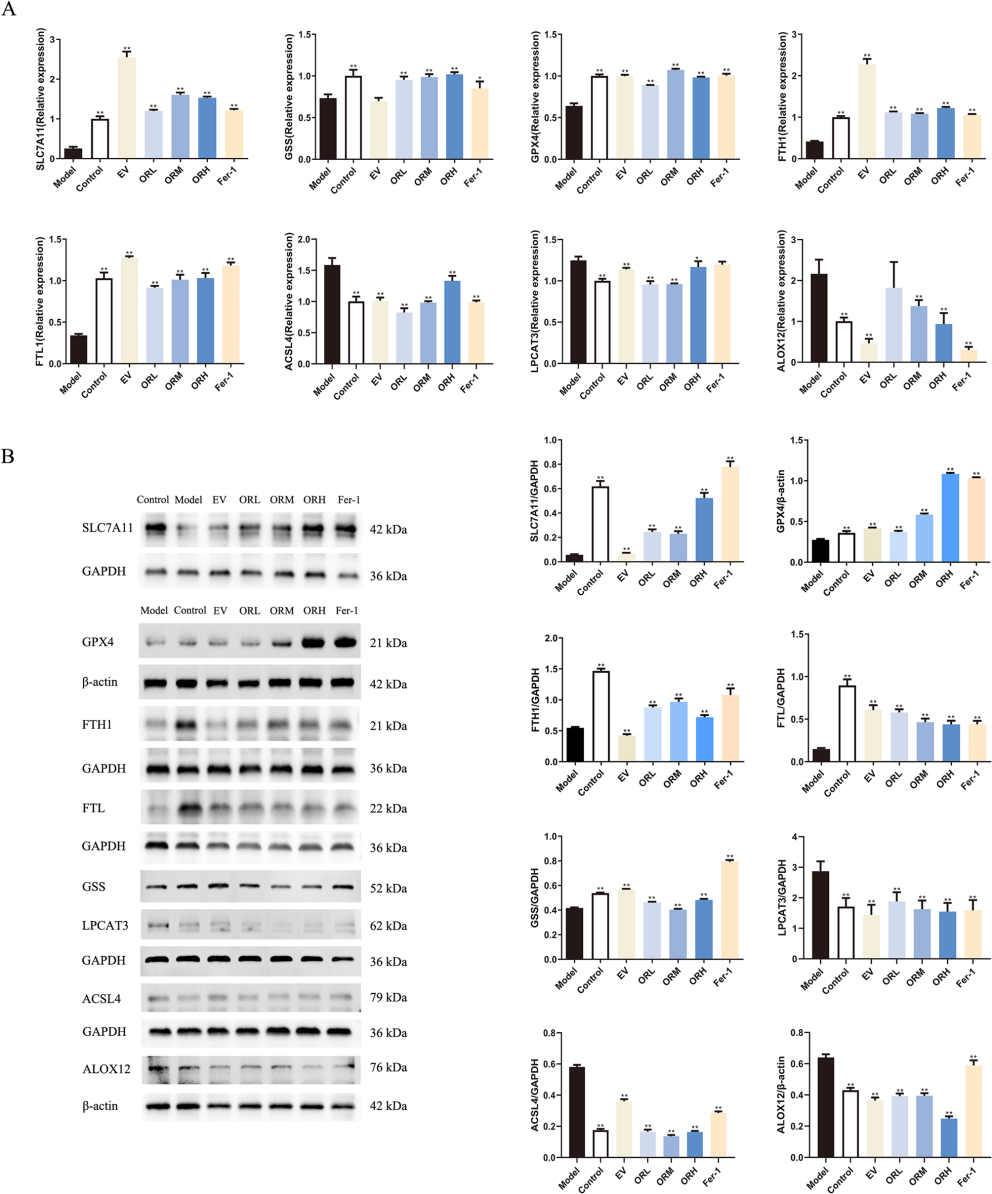
OR-containing serum regulates ferroptosis-related marker expression in OGCs. **(A)** mRNA and **(B)** protein expression of SLC7A11, GSS, GPX4, FTH1, FTL, ACSL4, LPCAT3, and ALOX12. Results are expressed as mean ± SD for each group (*n* = 3). *P < 0.05, **P < 0.01 compared with the model group

The expression of the Fe storage regulators FTH1 and FTL was evaluated in erastin-stimulated OGCs using RT-qPCR and western blotting. Erastin stimulation downregulated FTH1 and FTL expression, whereas OR-containing serum markedly upregulated it, confirming the mitigation of ferroptosis using OR-containing serum. Similar to the in vivo results, OR-containing serum promoted SLC7A11, GSS, and GPX4 expression in the erastin-stimulated OGCs, contributing to GSH production. Furthermore, OR-containing serum significantly inhibited the expression of ACSL4, LPCAT3, and ALOX12 (Fig. 9A and B). These results indicated that OR-containing serum inhibits ferroptosis through the GPX4/ACSL4 axis in erastin-treated OGCs.

## Discussion

Ovarian aging is a common cause of decreased ovarian function and oocyte quality, impeding infertility treatment and remaining a significant clinical concern. In this study, a rat model of D-galactose-induced ovarian aging was used to investigate the therapeutic effect of OR on ovarian aging. The results indicated that OR can partially restore ovarian function and increase follicle number. Furthermore, OR can alleviate excessive ovarian accumulation of Fe and cell apoptosis, reduce lipid peroxidation attributed to the accumulation of MDA and 4-HNE, increase the activities of antioxidant enzymes (GPX4 and GSH), inhibit ferroptosis, and delay ovarian aging. This mechanism was confirmed in erastin-stimulated OGCs, as evidenced by the altered expression of markers of ferroptosis regulation; that is, SLC7A11, GSS, GPX4, ACSL4, LPCAT3, ALOX12, FTH1, and FTL, and reduced Fe accumulation.

The ovarian aging process is significantly faster than that of the external genital organs and testes [27], with its terminal phase marked by menopause. The age-related reduction in ovarian follicles induces menstrual cycle disorder and, ultimately, cessation of menstruation. Moreover, deteriorated oocyte quality gradually decreases fertility [28]. Mechanistically, weakened negative feedback due to decreased E_2_ concentration in the hypothalamic and adenohypophyseal axes increases FSH and decreases AMH levels [29]. As females age, the capacity of the antioxidant system also diminishes, causing a gradual accumulation of ROS and increased oxidative stress-induced cell damage within the ovaries, hastening ovarian aging [30]. Hence, potential strategies for enhancing ovarian hypofunction include mitigating lipid peroxides, eliminating free radicals, and optimizing mitochondrial respiration. Although various therapeutic interventions and clinical measures are directed toward these objectives, further exploration is necessary to establish effective treatments.

TCM has a long, significant history, characterized by rich traditional functionalities and refined formulation principles. The complex composition of TCM formulations is a challenge in the continued development of TCM [31]. Several TCM formulations have been shown to prevent ovarian aging through their antioxidative and anti-aging properties [32]. OR, one such TCM, primarily comprises proteins, amino acids, fatty acid esters, steroid hormones, phospholipids, fat-soluble vitamins, nucleic acids, steroids, and trace elements [33]. Its diverse composition confers various biological functions, including antioxidant, free radical scavenging, and anti-aging effects [34, 35]. OR exerts antioxidant and anti-aging effects by elevating the levels of antioxidant enzymes, including SOD and its subtype copper-zinc-SOD (SOD1), peroxidase, and glutathione peroxidase, increasing total antioxidant capacity, and decreasing MDA levels in the serum, brain, and liver tissues of elderly female rats [36]. Our previous study revealed that the compound Yifuning Soft Capsule, which contains OR as the primary ingredient, effectively increases serum E_2_ levels in menopausal model rats. Furthermore, it reduces FSH and LH levels, enhances various neurotransmitters, hormones, cytokines, and their receptors in the hypothalamus–pituitary–gonadal axis, stabilizes the imbalance of hypothalamic monoamine neurotransmitters, and ameliorates histopathological changes in ovarian and uterine tissues, delaying reproductive aging [37]. However, further investigation was then required to elucidate the molecular mechanisms through which OR delays ovarian aging.

In this study, D-galactose was used to establish an ovarian aging model. The assessment of serum hormone levels and examination of ovarian pathological morphology confirmed the successful establishment of the model. The ovaries of the ovarian aging model rats were well-developed, and follicles of all grades were visible after OR treatment. Moreover, OR supplementation elevated the levels of E_2_ and AMH and diminished those of FSH and LH, improving ovarian endocrine and reserve functions and delaying pathological changes in D-galactose-induced ovarian aging rats. Hence, OR remarkably ameliorated ovarian injury during D-galactose-induced ovarian aging.

The mechanism through which OR protects ovarian function has been evaluated. Evidence indicates that Fe accumulation is associated with menopausal symptoms [38]. Excessive aggregation of Fe causes irregularities in the hypothalamic–pituitary–ovarian axis in rats [39]. Non-heme Fe aggregation in the ovarian tissue may be linked to ovarian aging due to heightened oxidative stress [40]. Fe accumulation contributes to age-related oxidative damage and causes various types of programmed cell death, including ferroptosis, apoptosis, and mitophagy. Therefore, the role of Fe overload in ovarian aging varies. Some previous studies have shown that oxidative stress could have a feedback impact, modulating the expression of genes related to maintaining Fe balance and altering intracellular Fe^2+^ concentration [41]. In addition, excess Fe^2+^ may trigger ROS production through the Fenton reaction, causing ferroptosis. This process indicates an intricate correlation between ferroptosis and oxidative stress, indicating that ferroptosis is a crucial element in oxidative stress-induced damage [42]. Accordingly, pharmacological suppression of ferroptosis could represent a promising therapeutic target for treating ovarian aging. Indeed, OR can increase SOD activity and reduce MDA content in the ovaries and uteri of aged female mice, enhancing the antioxidant effect of the reproductive organs and improving the degree of atrophy and aging [21]. Similarly, OR has a protective effect on OGCs damaged by oxidative stress [22]. However, whether OR prevents ovarian aging and injury by inhibiting ferroptosis remains unclear.

The induction of ferroptosis relies on Fe. Ferroptosis is intricately related to lipid peroxidation, which disrupts the balance of the biological redox system and serves as a major driving force of this process. Disruption of the phospholipid bilayer structure of cell membranes and the cytotoxic effects induced by lipid peroxidation products, such as MDA and 4-HNE, can initiate ferroptosis [43]. Notably, the endogenous antioxidant enzymes GPX4 and GSH can convert lipid peroxides into non-toxic lipids, helping resist lipid peroxidation and inhibiting ferroptosis. To verify these ferroptotic characteristics during ovarian aging and determine whether OR can regulate ferroptosis to protect ovarian function, the protective effects of OR on ovarian cells in D-galactose-induced ovarian aging in rats were evaluated in this study. Our data show that OR can protect against ovarian injury and inhibit ferroptosis. In a rat model of D-galactose-induced ovarian aging, Fe accumulation increased along with levels of lipid peroxidation products (MDA and 4-HNE) and the number of apoptotic cells, whereas antioxidant (GSH and GPX4) levels and the number of proliferating cells decreased. Following intervention with OR and EV, Fe accumulation, apoptotic cell abundance, and 4-HNE expression levels in the ovarian tissue decreased, while proliferating cells and GPX4 expression levels increased. Serum MDA levels also decreased significantly with reduced serum GSH consumption. These results indicate that D-galactose can induce ovarian aging and ferroptosis in rats, whereas OR reduces the accumulation of Fe and lipid peroxide products in the ovaries, restores the function of the antioxidant system, stimulates cell proliferation, inhibits ovarian ferroptosis, and reduces the degree of atrophy and aging. Similarly, OR-containing serum intervention markedly increased the viability of erastin-stimulated OGCs, inhibited cell death, reduced Fe accumulation and lipid peroxidation production, increased antioxidant capacity, and maintained mitochondrial homeostasis.

Given the proven therapeutic efficacy of OR in ovarian aging, elucidating its mechanism of action is critical. Ferroptosis is linked to the modulation of multiple key genes that affect cellular pathophysiological mechanisms [44]. For instance, Fe storage is pivotal in protecting cells from the oxidative stress caused by excess redox-imbalanced free Fe [45]. Ferritin, comprising FTH and FTL, serves as the primary intracellular site for Fe storage [46]. Moreover, mitochondrial ferritin exhibits significant similarity to FTH and acts as a crucial protein for Fe storage within the mitochondria [47]. Aberrant upregulation of transferrin and FTH/FTL chain expression has been observed in naturally aging rat ovaries. In this study, treatment with OR markedly diminished intracellular Fe levels, partially regulating FTH1 and FTL expression in OGCs and D-galactose-induced ovarian aging in rats.

Furthermore, RNA-Seq identified genes and pathways associated with the intervention of OR in ovarian aging. Compared to the D-galactose model, 2,975, 878, and 2,768 DEGs were identified in the control, EV, and OR groups, respectively. Further comparative analysis of the RNA-Seq datasets across these groups revealed that DEGs were enriched in pathways including lipid metabolism, ovarian steroidogenesis, PI3K/Akt signaling, glutathione metabolism, and ferroptosis, indicating the potential regulatory effect of OR on ferroptosis.

The enrichment analysis of the ferroptosis signaling pathway drew particular attention. To further validate these findings, western blotting and RT-qPCR techniques were used to assess the expression levels of SLC7A11, GSS, GPX4, ACSL4, LPCAT3, and ALOX12 genes and proteins following OR intervention in ovarian aging rats. D-galactose or erastin combined with OR treatment restored the expression of SLC7A11, GSS, and GPX4 and inhibited the accumulation of ACSL4, LPCAT3, and ALOX12. Since GPX4 is a pivotal protein involved in ferroptosis, its upregulation is crucial in blocking this process. GPX4 uses GSH to impede lipoxygenase-induced lipid peroxidation by diminishing hydrogen peroxide levels in phospholipids [48]. The xCT antiporter system, composed of SLC7A11 and SLC3A2, regulates intracellular GSH levels by promoting cystine uptake and glutamate export [49]. In addition, inactivation of the ACSL4/LPCAT3/ALOX12 axis is associated with modulating ferroptosis sensitivity by catalyzing polyunsaturated fatty acid metabolism and influencing cellular lipid composition [50]. These findings indicate that OR may protect OGCs and ovarian tissue from ferroptosis via modulation of the GPX4/ACSL4 signaling pathway. The RT-qPCR and western blotting results aligned with the RNA-Seq data, further confirming that OR regulates the ferroptosis pathway. However, given the complex interplay between oxidative stress and ferroptosis, the detailed mechanism of OR against oxidative stress in aging rats and OGCs requires further investigation.

In this study, the D-galactose-induced ovarian aging rat model served as a valuable yet imperfect experimental tool. This model is most appropriately used as a complementary and preliminary tool for investigating specific aging-related mechanisms—such as oxidative stress—instead of as a comprehensive representation of human ovarian aging. This limitation is because the D-galactose-induced ovarian aging does not fully replicate the complex, multifactorial, and systemic characteristics of natural physiological ovarian aging. Therefore, more extensive research using natural aging models is required before clinical applications can be considered.

Another limitation of this study is that it did not identify the critical components responsible for the beneficial effects of OR. This challenge is compounded by the fact that the therapeutic efficacy of OR likely depends on the synergistic actions of multiple constituents instead of individual compounds. Consequently, further investigation is required to elucidate the mechanisms through which the components of OR modulate ovarian aging.

## Conclusions

In this study, OR significantly inhibited ferroptosis by mediating the GPX4/ACSL4 pathway, thereby partially improving ovarian function and delaying ovarian aging at the tissue level. Moreover, OR-containing serum regulates the GPX4/ACSL4 pathway, reduces the levels of Fe, ROS, 4-HNE, and lipid peroxidation in OGCs, increases antioxidant capacity, maintains mitochondrial homeostasis, enhances OGC proliferation, and inhibits OGC ferroptosis. Restoring ovarian function is highly significant in treating ovarian aging, with the potential to enhance fertility and the overall reproductive health of females. Importantly, OR may offer a promising alternative for individuals with ovarian aging for whom HRT is contraindicated or who are reluctant to use it. OR shows therapeutic potential and is promising for preventing and treating ovarian aging.

## Supplementary Information

Supplementary Material 1.

Supplementary Material 2.

## Data Availability

The data that support the findings of this study are available from the corresponding author upon reasonable request.

## Abbreviations

4-HNE: 4-hydroxynonenal
ACSL4: acyl-CoA synthetase long chain family member 4
ALOX12: arachidonate 12-lipoxygenase
AMH: anti-müllerian hormone
CCK-8: cell counting kit-8
DAPI: 4′,6-diamidino-2-phenylindole
DCFH-DA: dichloro-dihydro-fluorescein diacetate
E_2_: Estradiol
ELISA: enzyme-linked immunosorbent assay
EV: estradiol valerate
FBS: fetal bovine serum
Fer-1: Ferrostatin-1
FSH: follicle-stimulating hormone
FTH1: ferritin heavy chain 1
FTL: ferritin light chain
GPX4: glutathione peroxidase 4
GSH: glutathione
GSS: glutathione synthetase
HRT: hormone replacement therapy
IF: immunofluorescence
LH: luteinizing hormone
LPCAT3: lysophosphatidylcholine acyltransferase 3
MDA: malondialdehyde
MMP: mitochondrial membrane potential
OGC: ovarian granulosa cell
OR: *Oviductus Ranae*
ORH: high dose OR group
ORL: low dose OR group
ORM: medium dose OR group
P: progesterone
PCNA: proliferating cell nuclear antigen
PCOS: polycystic ovary syndrome
RT-qPCR: reverse transcription-quantitative polymerase chain reaction
ROS: reactive oxygen species
SD: Sprague–Dawley
SLC7A11: solute carrier family 7 member 11
SOD: superoxide dismutase
TCM: traditional Chinese medicine
TMRE: tetramethylrhodamine ethyl ester perchlorate
TUNEL: terminal deoxynucleotidyl transferase dUTP nick end labeling
