# Supplementary figures and images for: Oviductus Ranae alleviates D-galactose-induced ovarian aging by inhibiting ferroptosis and regulating the GPX4/ACSL4 pathway
Source: J Ovarian Res. 2025 Nov 28;19:8. doi: 10.1186/s13048-025-01857-2 (PMC12781486; doi:10.1186/s13048-025-01857-2)

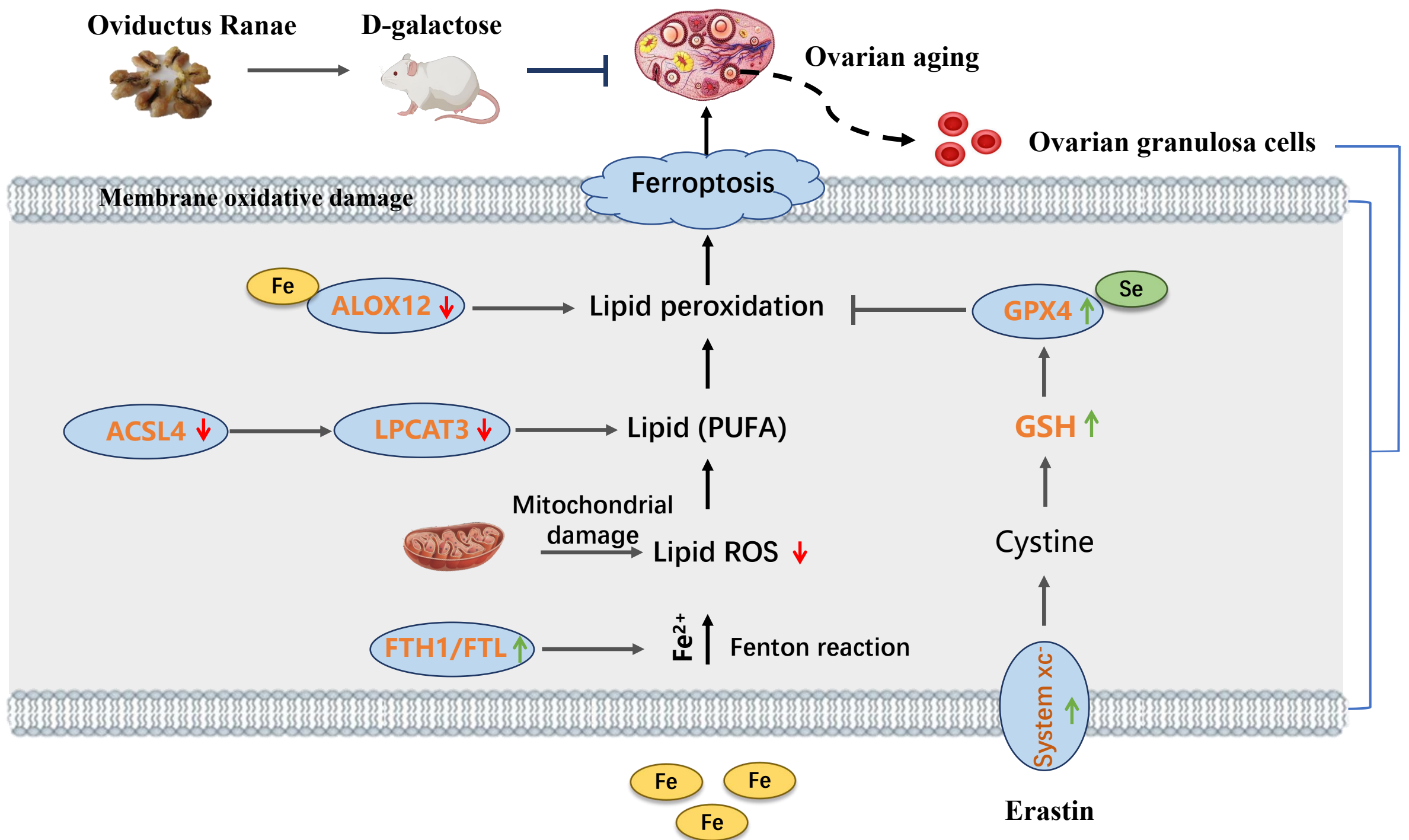

Supplement: Supplementary file 1 — Supplementary Material 1. [file 13048_2025_1857_MOESM1_ESM.pdf]
