# Supplementary material for: Oviductus Ranae alleviates D-galactose-induced ovarian aging by inhibiting ferroptosis and regulating the GPX4/ACSL4 pathway
Source: J Ovarian Res. 2025 Nov 28;19:8. doi: 10.1186/s13048-025-01857-2 (PMC12781486; doi:10.1186/s13048-025-01857-2)

Fig. 5B SLC7A11 GPX4 ALOX12 β-actin

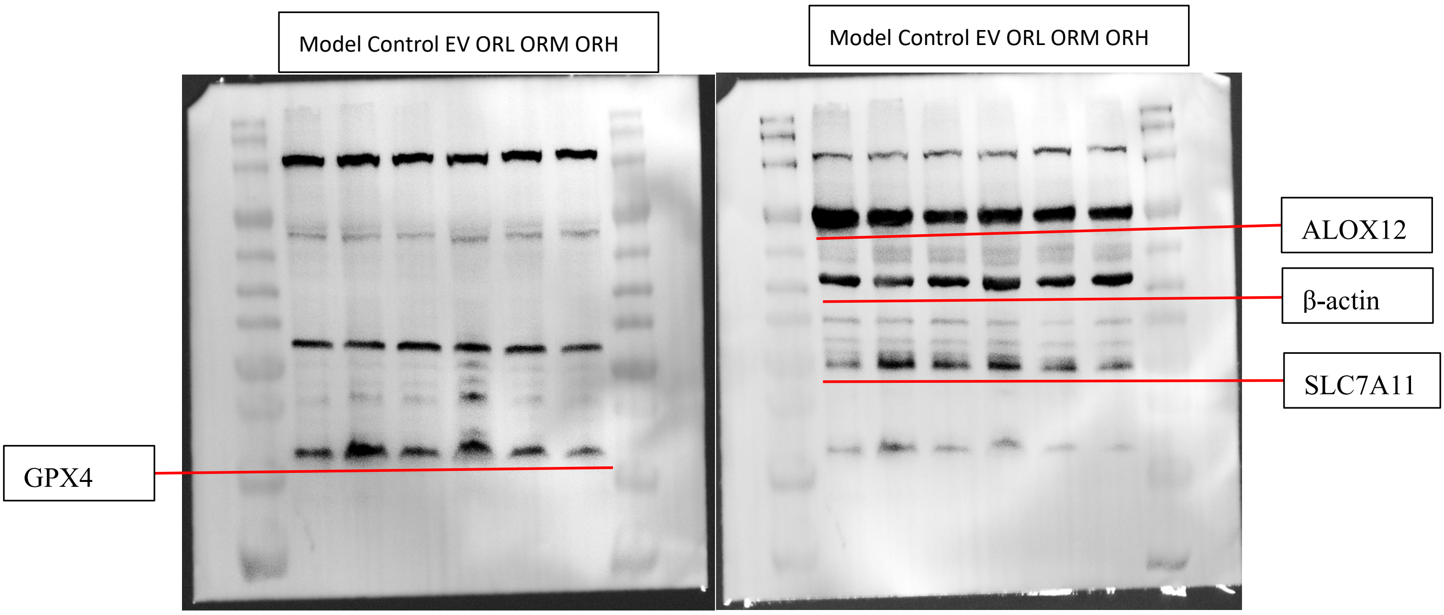

Fig. 5B GSS GAPDH

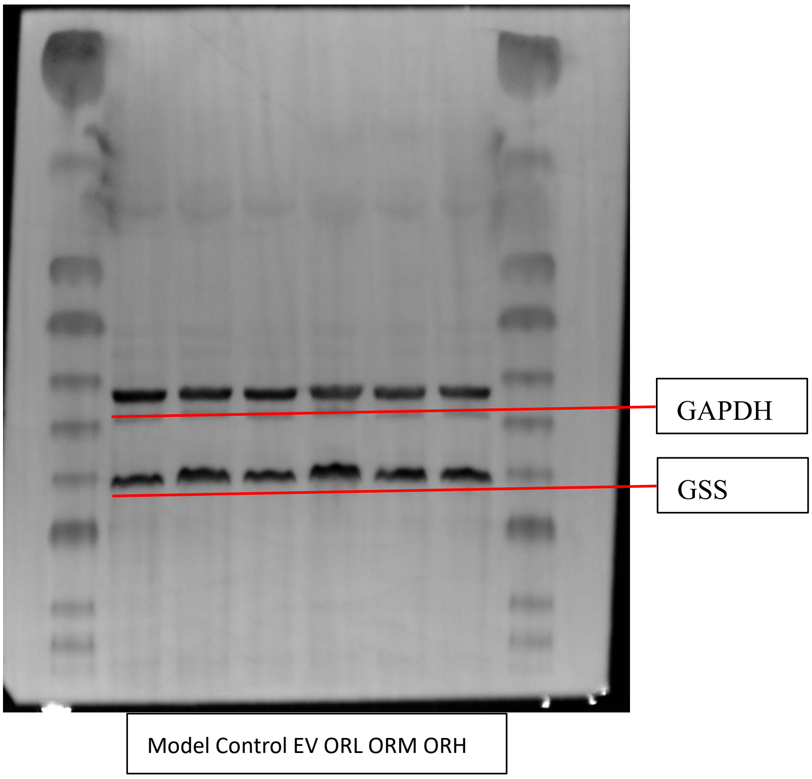

Fig. 5B FTL β-actin

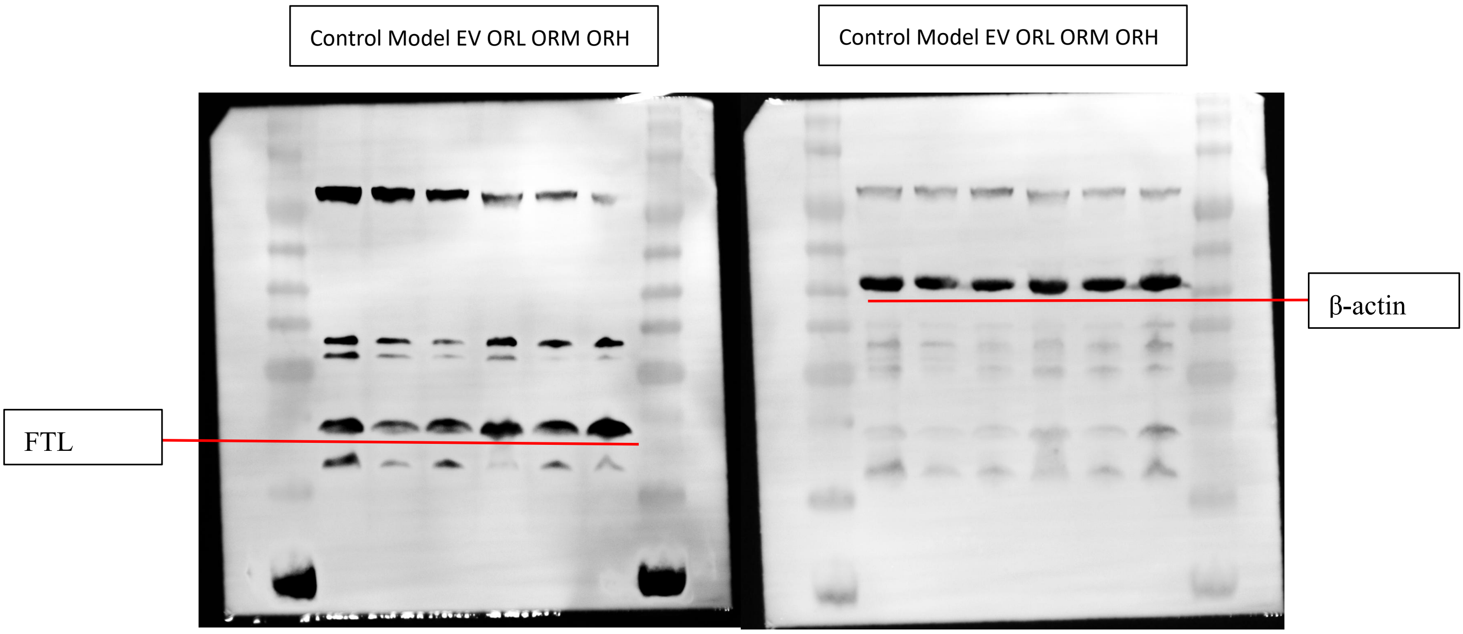

Fig. 5B FTH1 β-actin

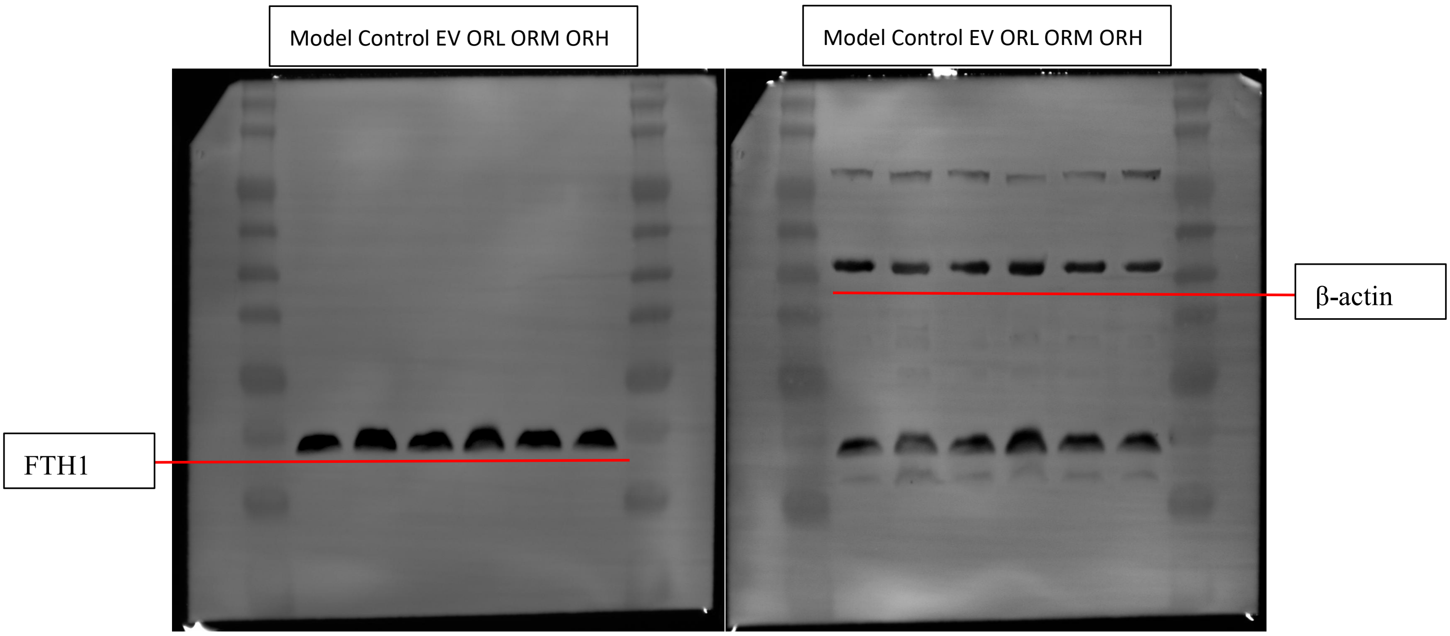

Fig. 5B ACSL4 β-actin

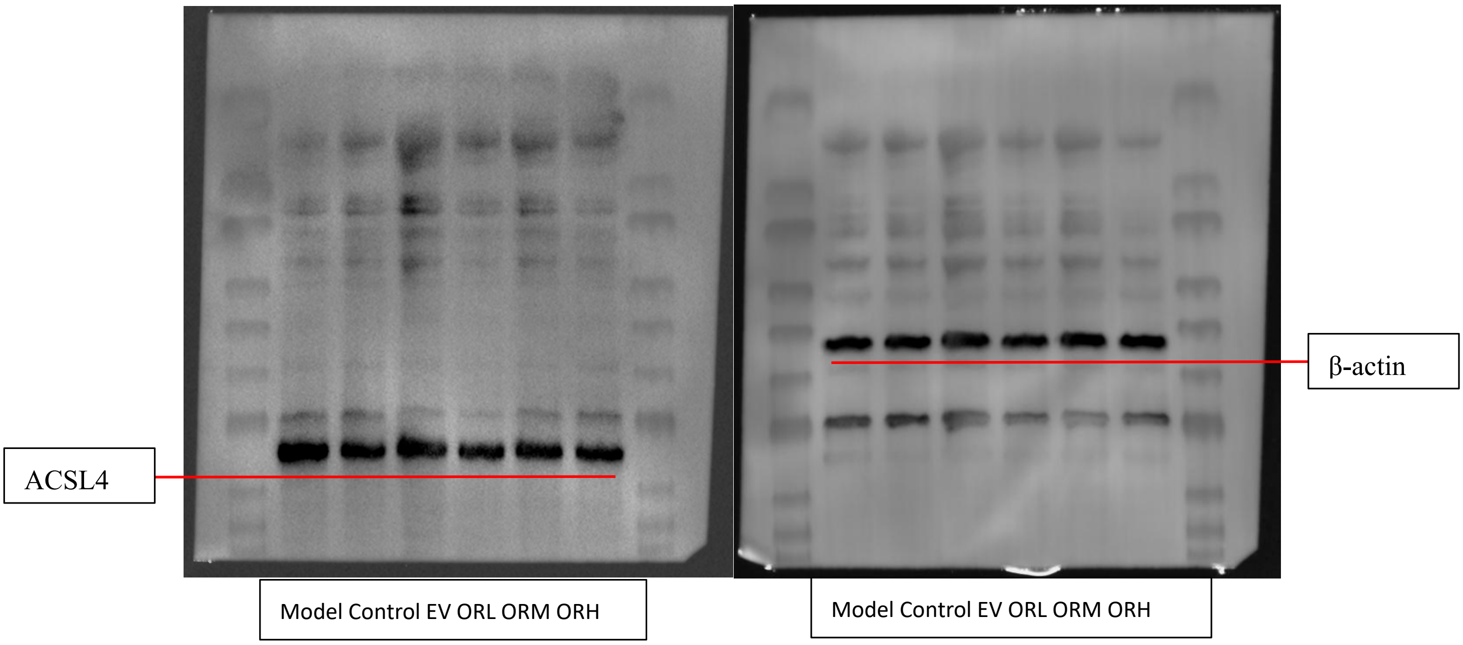

Fig. 5B LPCAT3 GAPDH

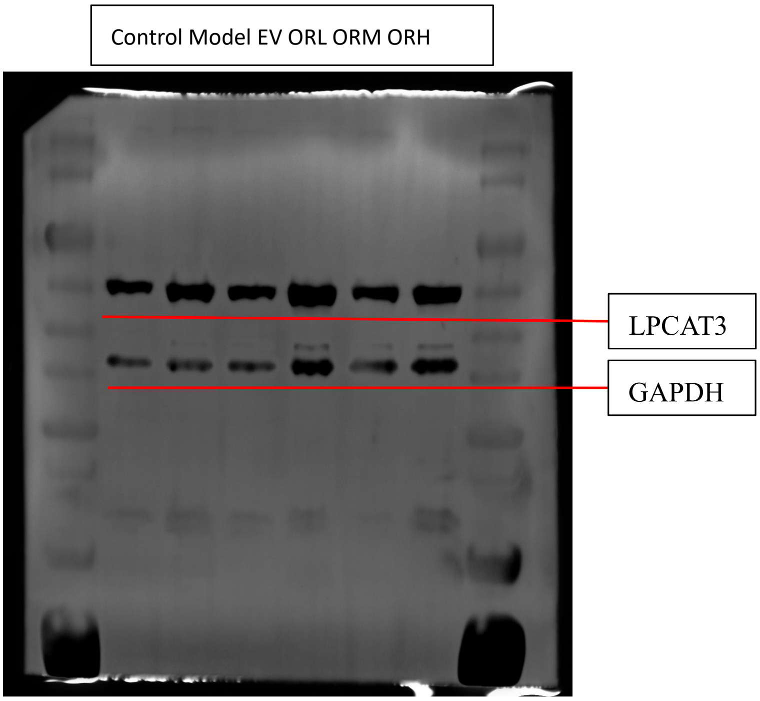

Fig. 9B SLC7A11 GAPDH

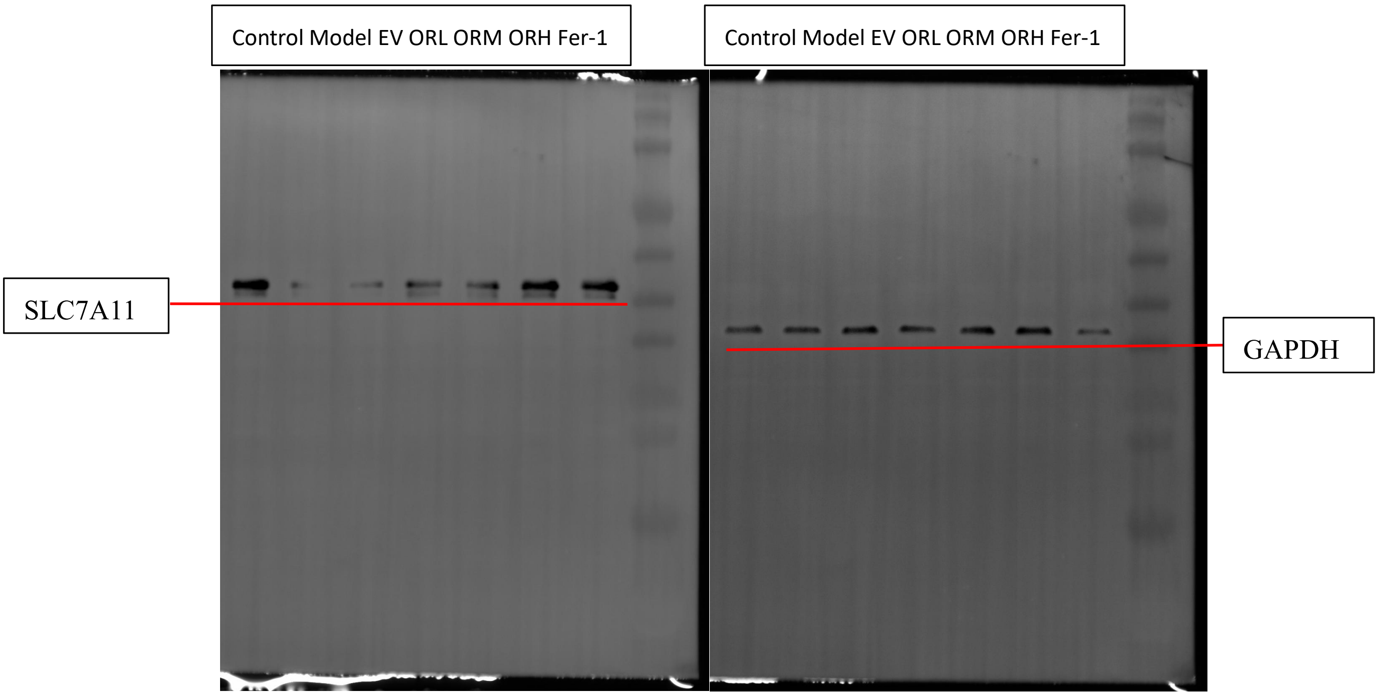

Fig. 9B GPX4 β-actin

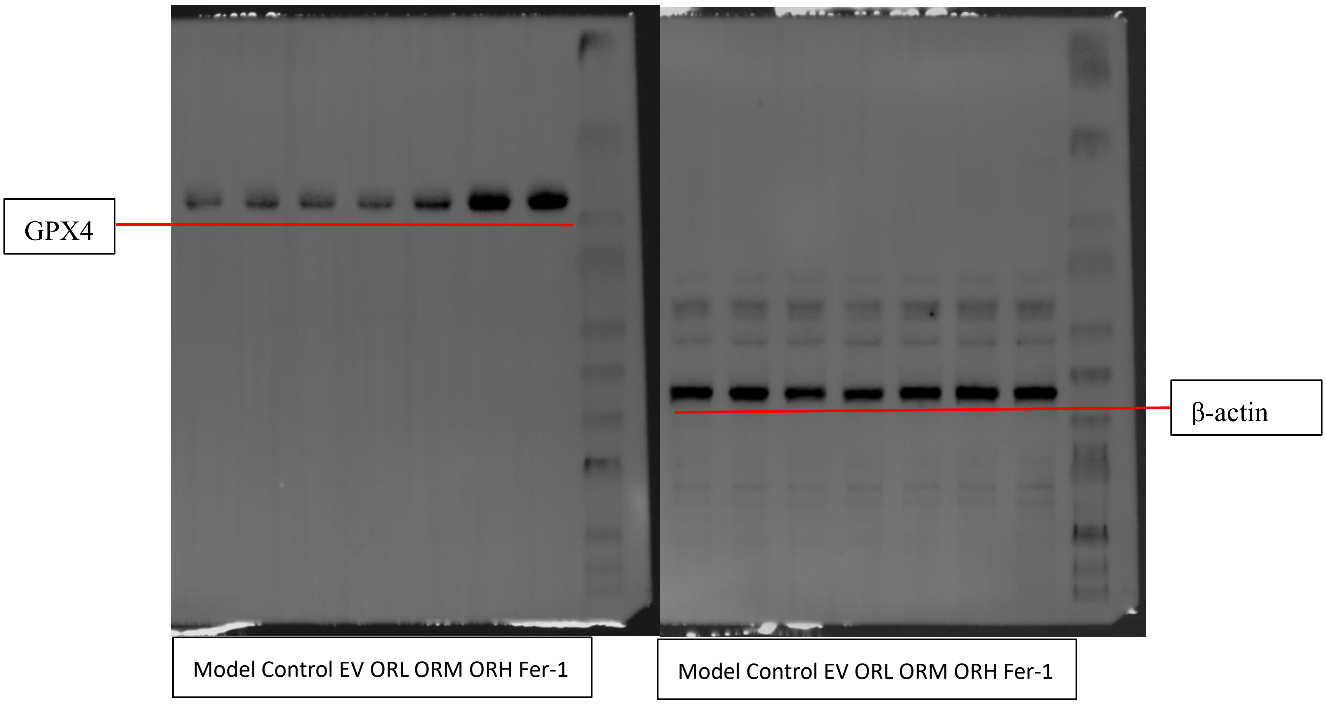

Fig. 9B FTH1 GAPDH

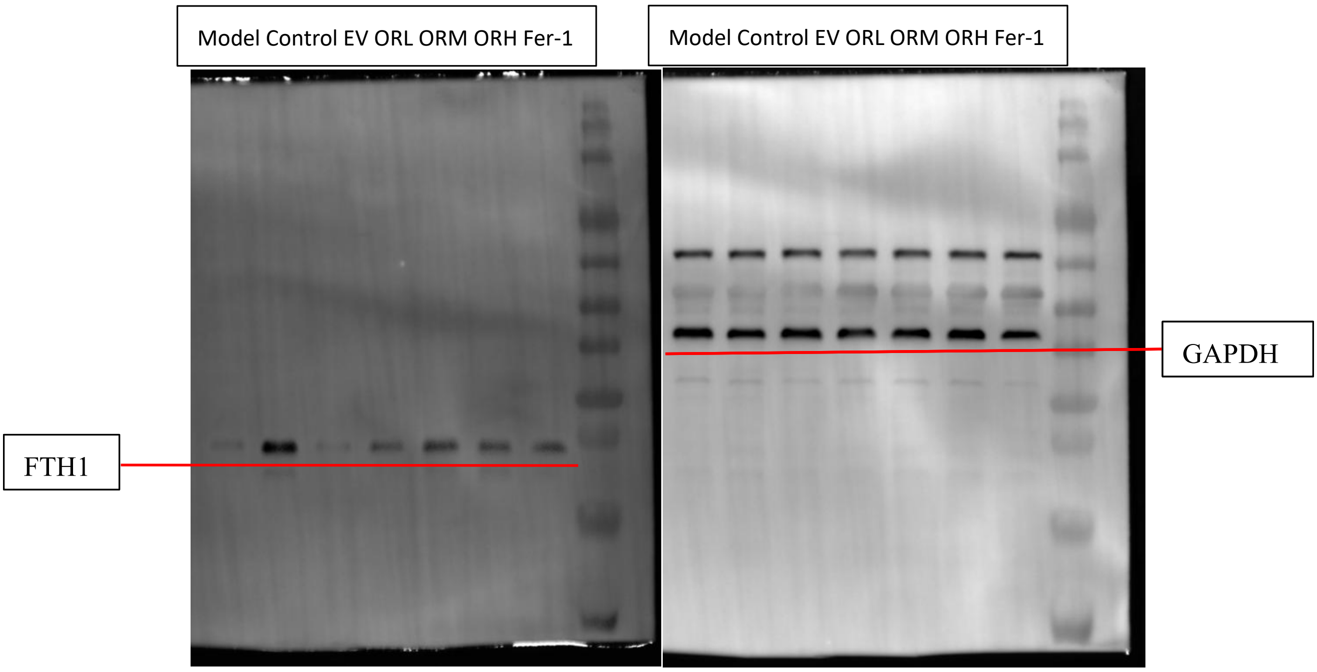

Fig. 9B FTL GAPDH

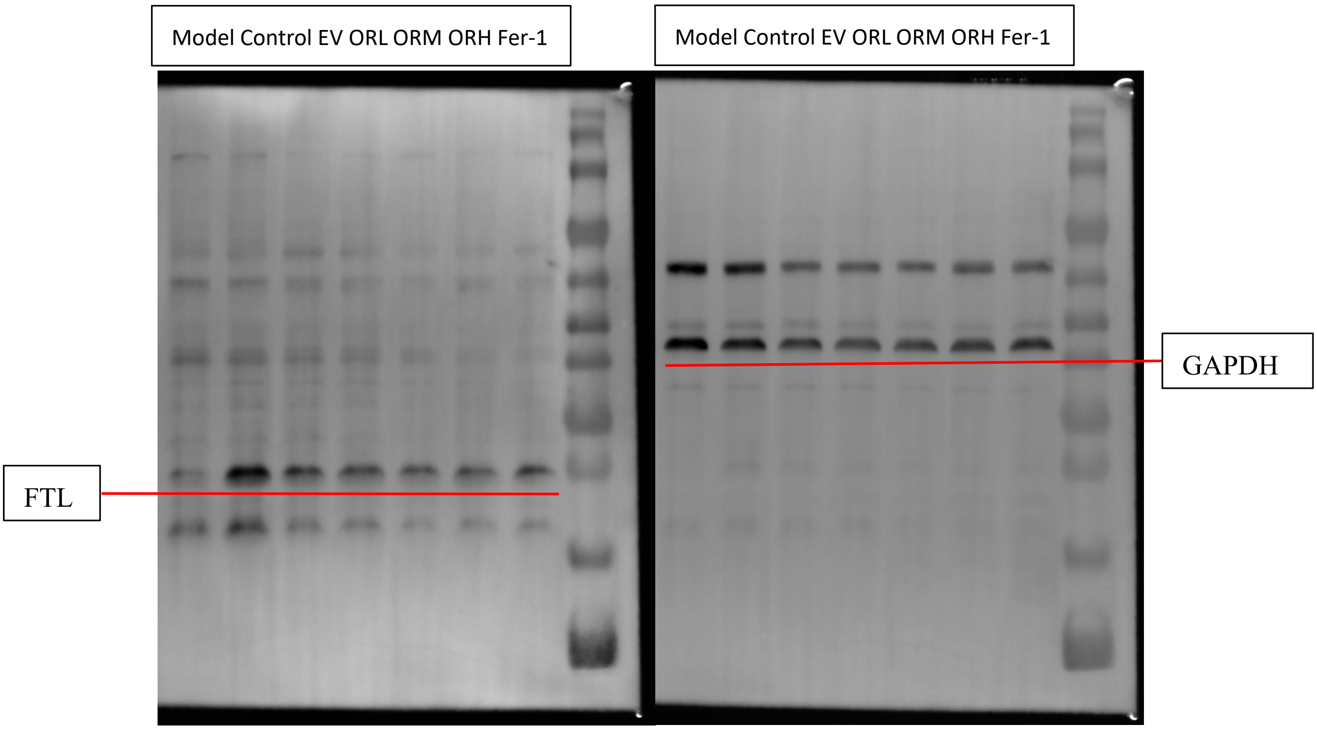

Fig. 9B GSS LPCAT3 GAPDH

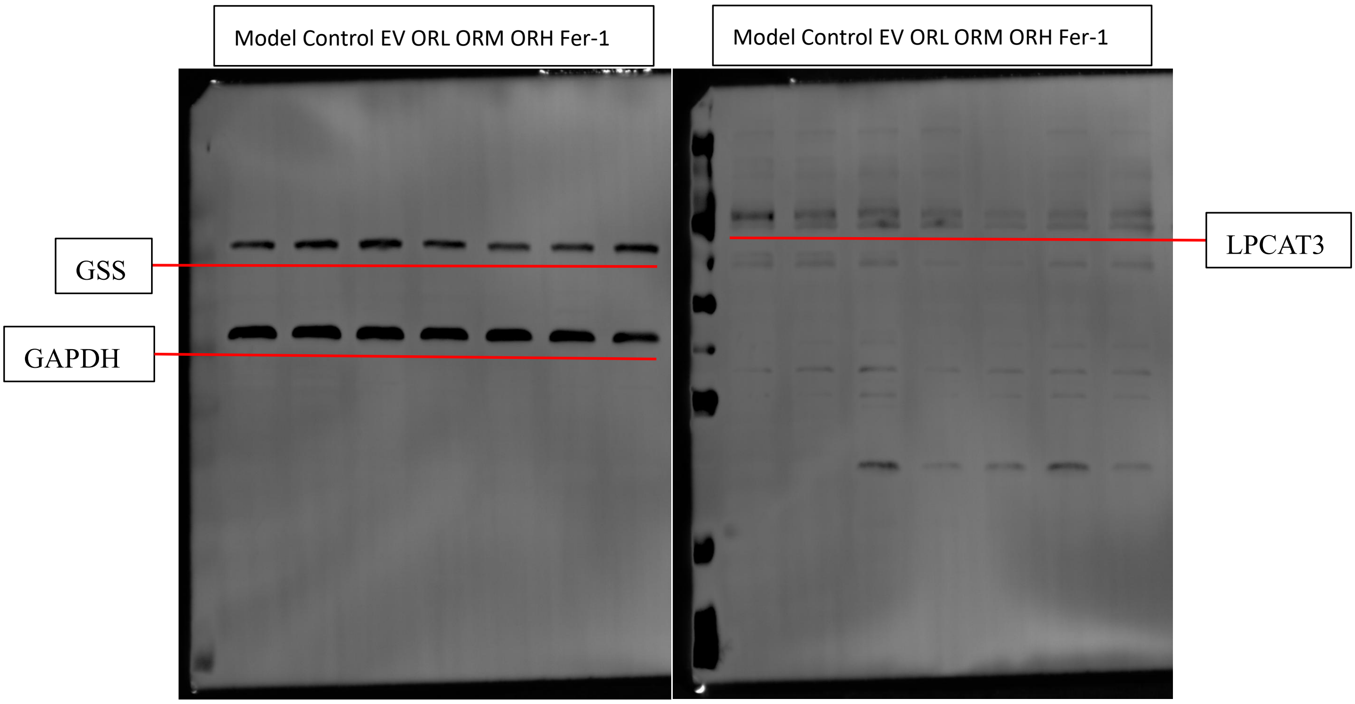

Fig. 9B ACSL4 GAPDH

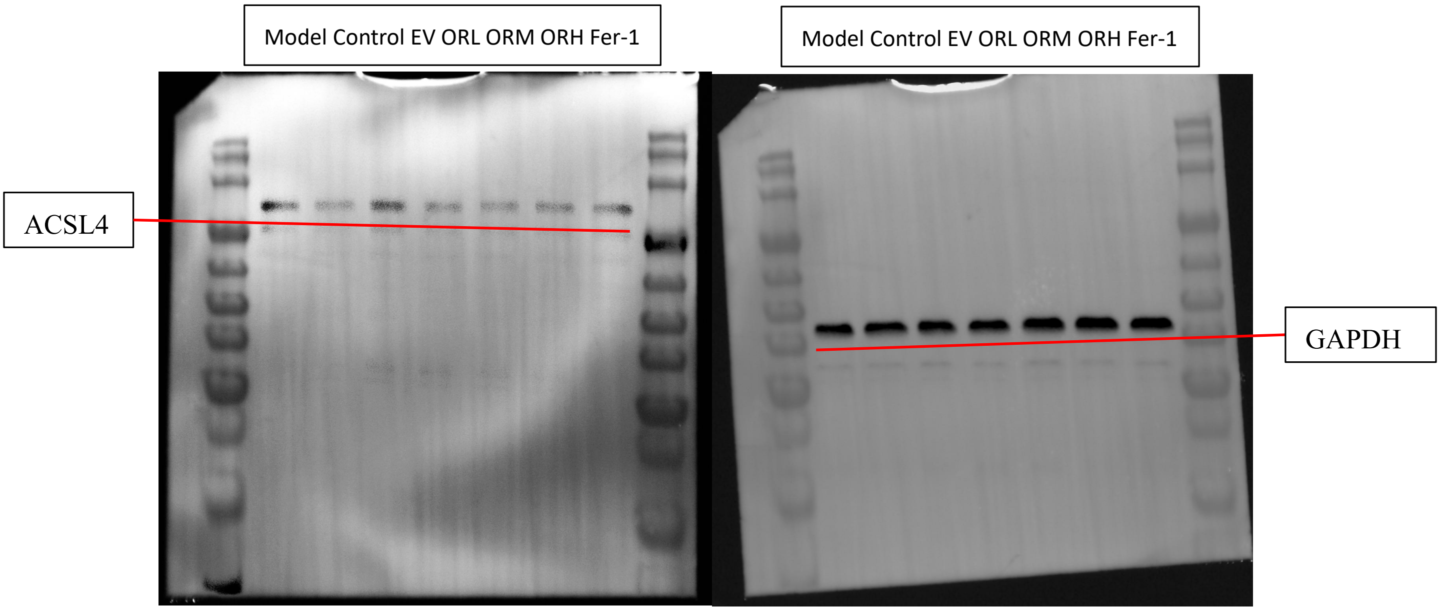

Fig. 9B   ALOX12   β-actin

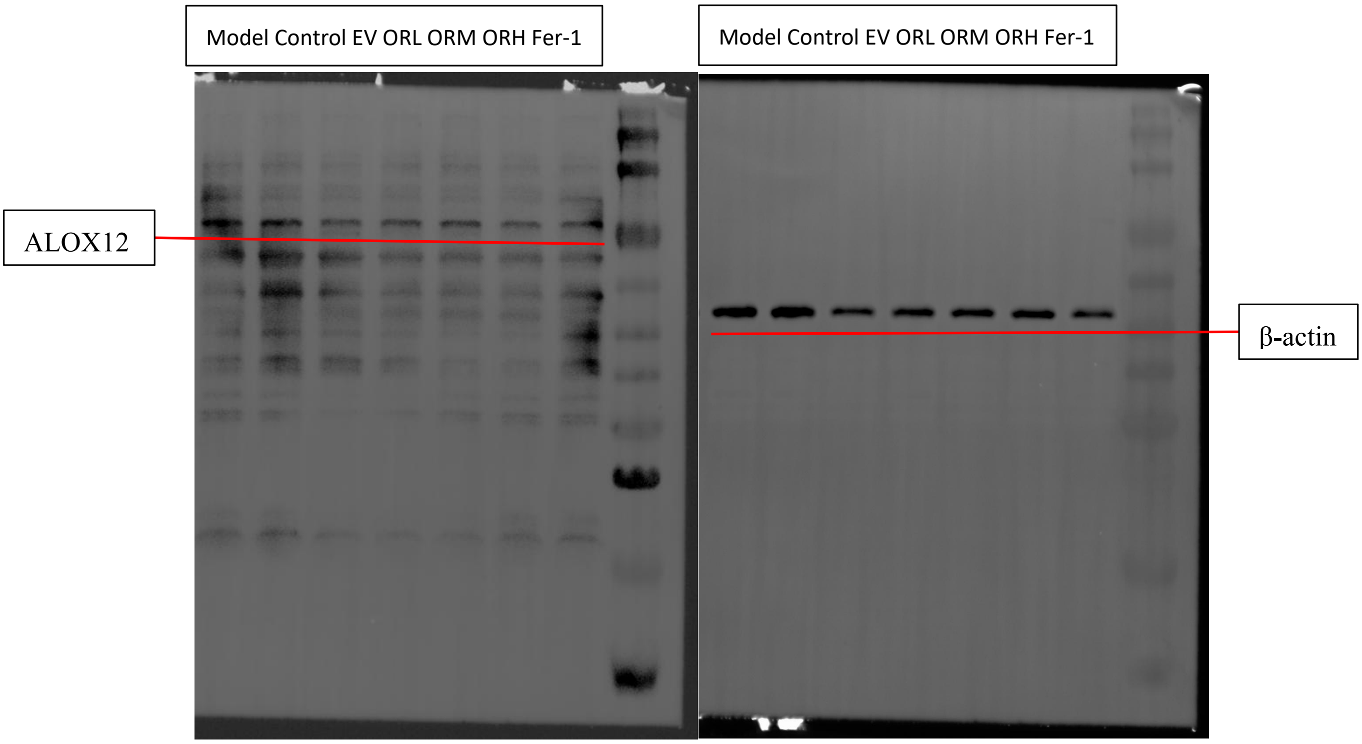

Supplement: Supplementary file 2 — Supplementary Material 2. [file 13048_2025_1857_MOESM2_ESM.pdf]
